# *Lactobacillus murinus* alleviate intestinal ischemia/reperfusion injury through promoting the release of interleukin-10 from M2 macrophages via Toll-like receptor 2 signaling

**DOI:** 10.1186/s40168-022-01227-w

**Published:** 2022-03-03

**Authors:** Jingjuan Hu, Fan Deng, Bingcheng Zhao, Zebin Lin, Qishun Sun, Xiao Yang, Mei Wu, Shida Qiu, Yu Chen, Zhengzheng Yan, Sidan Luo, Jin Zhao, Weifeng Liu, Cai Li, Ke Xuan Liu

**Affiliations:** Department of Anesthesiology, Nanfang Hospital, Southern Medical University, Guangzhou, 1838 Guangzhou Avenue North, Guangzhou, 510515 China

**Keywords:** Intestinal ischemia/reperfusion injury, Gut microbiota, *Lactobacillus murinus*, Macrophages, Toll-like receptor 2, Interleukin-10

## Abstract

**Background:**

Intestinal ischemia/reperfusion (I/R) injury has high morbidity and mortality rates. Gut microbiota is a potential key factor affecting intestinal I/R injury. Populations exhibit different sensitivities to intestinal I/R injury; however, whether this interpopulation difference is related to variation in gut microbiota is unclear. Here, to elucidate the interaction between the gut microbiome and intestinal I/R injury, we performed 16S DNA sequencing on the preoperative feces of C57BL/6 mice and fecal microbiota transplantation (FMT) experiments in germ-free mice. The transwell co-culture system of small intestinal organoids extracted from control mice and macrophages extracted from control mice or Toll-like receptor 2 (TLR2)-deficient mice or interleukin-10 (IL-10)-deficient mice were established separately to explore the potential mechanism of reducing intestinal I/R injury.

**Results:**

Intestinal I/R-sensitive (Sen) and intestinal I/R-resistant (Res) mice were first defined according to different survival outcomes of mice suffering from intestinal I/R. Fecal microbiota composition and diversity prior to intestinal ischemia differed between Sen and Res mice. The relative abundance of *Lactobacillus murinus* (*L. murinus*) at the species level was drastically higher in Res than that in Sen mice. Clinically, the abundance of *L. murinus* in preoperative feces of patients undergoing cardiopulmonary bypass surgery was closely related to the degree of intestinal I/R injury after surgery. Treatment with *L. murinus* significantly prevented intestinal I/R-induced intestinal injury and improved mouse survival, which depended on macrophages involvement. Further, in vitro experiments indicated that promoting the release of IL-10 from macrophages through TLR2 may be a potential mechanism for *L. murinus* to reduce intestinal I/R injury.

**Conclusion:**

The gut microbiome is involved in the postoperative outcome of intestinal I/R. *Lactobacillus murinus* alleviates mice intestinal I/R injury through macrophages, and promoting the release of IL-10 from macrophages through TLR2 may be a potential mechanism for *L. murinus* to reduce intestinal I/R injury. This study revealed a novel mechanism of intestinal I/R injury and a new therapeutic strategy for clinical practice.

Video Abstract.

**Supplementary Information:**

The online version contains supplementary material available at 10.1186/s40168-022-01227-w.

## Background

Intestinal ischemia-reperfusion (I/R) is a common but grave condition in some critical clinical settings such as acute mesenteric ischemia, hemorrhagic, or septic shock, severe burns, and specific surgical procedures, including cardiopulmonary bypass (CPB), small-bowel transplantation, and abdominal aortic surgery [1]. I/R not only causes local intestinal injury but can also disrupt the intestinal mucosal barrier [2], allowing enteric bacterial endotoxins to penetrate the blood and cause extraintestinal multiple organ dysfunction or even failure with high morbidity and mortality [3, 4]. Currently, the mechanisms underlying intestinal I/R injury are not understood fully, and effective approaches for its clinical application are still lacking.

The human gastrointestinal tract houses a vast and diverse intestinal microflora that provides nutrients and intrinsic immunity, regulates epithelial cell growth, and fundamentally affects human health and disease [5]. We have previously confirmed that intestinal I/R induces significant intestinal flora disorders and indicated that intestinal microbial metabolites play an important regulatory role in intestinal I/R injury [6]. In our previous study, we observed that some specific pathogen-free male C57BL/6J mice survived 5 days after intestinal I/R, whereas some mice died quickly within 1 h post-intestinal I/R. To better explore the above phenomenon, mice that died within 1 h after reperfusion were defined as intestinal I/R-sensitive (Sen) mice, while those that survived up to 5 days were defined as intestinal I/R-resistant (Res) mice. Although these mice had the same genetic background, but their sensitivity to intestinal I/R injury significantly differed. We speculated that the different outcomes of mice suffering from intestinal I/R could be related to the differences in the gut microbiota. Further, we conducted 16S DNA sequencing and fecal microbiota transplantation (FMT) experiments to study the intestinal bacterial community and specific bacterial strains in the intestinal I/R model of mouse. The results showed that the relative abundance of *Lactobacillus murinus* (*L. murinus*) at the species level was higher in the Res than that in the Sen mice, suggesting that *L. murinus* could prevent from intestinal I/R injury. As a potential probiotic, *L. murinus* reportedly plays an important role in maintaining intestinal immune homeostasis in the mouse model of colitis by regulating T-lymphocyte activity [7]. In addition, *L. murinus* mediates anti-inflammatory effects in calorie-restricted mice [8]. Altered Schaedler flora contains *L.murinus*-colonized mice reduced leukocyte deposition in mesenteric I/R injury as compared to conventionally raised specific pathogen-free controls mice [9]. However, the role and mechanism of *L. murinus* in intestinal I/R injury remains unclear.

Macrophages belonging to the innate immune system exhibit a high degree of plasticity and can adapt their phenotype in response to environmental stimuli. Our earlier study reported that promoting the conversion of M1 macrophages to M2 may reduce intestinal I/R injury [10]. *Lactobacillus* plays an important role in intestinal immune function, upregulating T-lymphocyte activity and affecting the M1/M2 macrophage ratio [11]. *Lactobacillus reuteri* protects mice against *Salmonella typhimurium* challenge by triggering nitric oxide production in macrophages [12]. In addition, *L. plantarum* CLP-0611 ameliorates colitis in mice by polarizing M1 to M2-like macrophages [13]. These studies prompted us to investigate whether *L. murinus* plays a role in intestinal I/R injury through macrophages. *Lactobacillus murinus* is one of the most frequently found *Lactobacillus* strains; however, there are no reports on the interaction between *L. murinus* and macrophages.

Immune cells such as macrophages have been confirmed to recognize microorganisms through pattern recognition receptors including Toll-like receptors (TLRs) [14]. TLR2 and myeloid differentiation factor 88 (Myd88) are involved in identifying and signaling in response to a variety of gram-positive bacterium pathogen-associated molecular patterns by innate immune cells, particularly macrophages [15, 16]. *Lactobacillus reuteri* DSM 17938 plays a protective effect on experimental necrotizing enterocolitis via TLR2-mediated reduction of intestinal effector T-lymphocyte levels [17]. In addition, studies have shown that *L. casei* Lbs2 protects from experimental colitis through TLR2-dependent induction of T-regulatory response [18]. *Lactobacillus murinus* is a gram-positive bacterium that can be used as a ligand for TLR2 [17, 19], leading us to ponder whether *L. murinus* could reduce intestinal I/R injury through its interactions with TLR2. However, the role of *L. murinus* and TLR2 on macrophages in intestinal I/R injury has not been reported.

Interleukin-10 (IL-10) is an inflammatory and immunosuppressive factor that plays a vital role in controlling inflammation and preventing enteritis [20, 21]. The main sources IL-10 are monocytes, macrophages, and helper T cells. IL-10 production by TLR-activated macrophages is well documented. For instance, *Clostridium butyricum* directly triggered IL-10 production by intestinal macrophages in inflamed mucosa via the TLR2/MyD88 pathway, thereby preventing experimental colitis in mice [22]. Some findings indicated more severe arthritis in TLR2^−/−^ mice than wild-type (WT) controls and that TLR2^−/−^ mice promote the effector phase of arthritis through decreased IL-10 production by macrophages [23]. Some researchers used intestinal bacterial strains isolated from mice with colitis to stimulate murine peritoneal macrophages in vitro and found that IL-10 can be secreted from macrophages induced by *L. murinus* [24]. However, the role of IL-10 in I/R is currently controversial. IL-10 can prevent systemic and local acute inflammation after intestinal I/R [25]. Conversely, researchers found that IL-10 has not protective effect on intestinal I/R injury and increases tissue injury after selective intestinal I/R [26, 27]. Furthermore, the role of IL-10 secreted by macrophages during intestinal I/R injury is yet to be revealed.

Based on the above considerations, we hypothesized that the gut microbiome is associated with the postoperative outcome of intestinal I/R and that *L. murinus* alleviates intestinal I/R injury through macrophages, and promoting the release of IL-10 from macrophages through TLR2 may be a potential mechanism for *L. murinus* to reduce intestinal I/R injury. In this study, we investigated the relationship between the gut microbiome and postoperative outcome of intestinal I/R as well as the effects of *L. murinus* on intestinal I/R injury and elucidated the potential mechanisms. This study will shed light on a novel mechanism of intestinal I/R injury related to gut microbiota and provide a potential therapeutic strategy in clinical settings in the foreseeable future.

## Methods

### Animal experiments

All animal experimental procedures were carried out in accordance with the National Institutes of Health guidelines and were approved by the local Animal Care and Use Committee of the Nanfang hospital of Southern Medical University (Guangzhou, China) (approval number NFYY-2019-247). Six-to eight-week-old-specific pathogen-free male C57BL/6J mice were purchased from the animal center of Nanfang Hospital of Southern Medical University. TLR2^−/−^ and IL-10^−/−^ mice were purchased from Shanghai Model Organisms Center, Inc. Germ-free (GF) C57BL/6J mice were purchased from Cyagen Biosciences Company (Suzhou, China). All mice were housed under controlled temperature and humidity conditions with a 12-h light-dark cycle, had free access to food and water, and were fasted overnight before the experiment.

The model of intestinal I/R was established as described in our previous study [28, 29]. Briefly, mice were anesthetized with 4% isoflurane, and a non-invasive microvascular artery clip was placed on the superior mesenteric artery (SMA) for 60 min followed by clip removal for reperfusion. During the study period, body temperature was maintained at 37 °C with a heating pad, and liquid resuscitation was performed by subcutaneously injecting 0.5 ml of physiological saline just after reperfusion.

### Bacterial strains and growth conditions

*Lactobacillus murinus* freeze-dried powder (BeNa Culture Collection, Beijing, China) was dissolved with 0.5 ml Man Rogosa Sharpe (MRS) medium (HKM, Guangdong, China), then the bacteria liquid was coated on the blood plate, and bacterial colonies appeared after about 24 h. Single colonies were picked into MRS medium, were incubated at 37 °C under anaerobic conditions, and OD600 = 0.6–0.7 of cultures was measured until mid-log phase after 12–16 h of growth, at which time the colony count was 6.8 × 10^8^ CFU/mL by plate count. Frozen stocks of *L. murinus* (in MRS medium with 25% glycerol) were prepared, stored at − 80 °C for further experiments. Then, 50 μl frozen stocks of *L. murinus* were added to 5 ml MRS medium and incubated at 37 °C under aerobic conditions for 12 h, and then used for gavage of mice. In order to evaluate the total amount of *L. murinus* DNA in cecum and stool samples, it was quantified by quantitative real-time polymerase chain reaction (PCR) using the following primers: *L. murinus* [30], LactoM-F (5′-TCGAACGAAACTTCTTTATCACC-3′) and LactoM-R (5′-CGTTCGCCACTCAACTCTTT-3′).

### Animal experimental design

Feces from C57BL/6J mice (*n* = 70) were collected before intestinal I/R following which the mice underwent intestinal ischemia and the survival rate was observed after reperfusion. Mice that died within 1 h after reperfusion were defined as “Sen” mice (*n* = 7), while those that survived up to 5 days were defined as “Res” mice (*n* = 7). These mice were purchased from the same batch of the same manufacturer. Sen and Res mice were housed randomly in different cages. There was no difference in body weight and age of the mice. They were all 6–8-week-old C57BL/6J mice, weighing 20–25 g.

Mice were divided into the following experimental groups. Except that the number of experimental mice with survival rate is *n* = 20, the other mice experiment involves *n* = 5–8.

### FMT experiment

FMT was performed according to the modified method described previously [31]. Briefly, preoperatively collected feces of Sen and Res mice (donor mice) were resuspended in PBS at 0.125 g /ml. An amount of 0.1 ml of the solution was administered to GF mice (Receptor mice) in the corresponding groups orally via gastric gavage tube. The GF mice that received feces from Sen mice and Res mice were referred to as the Sen feces group and Res feces group (5–6 mice per group). All the mice had free access to food and water, and the gavage was performed in a sterile environment. Mice were performed intestinal ischemia 60 min and reperfusion 120 min surgery after 3 days of transplantation, and then blood, ileum, kidney, lung, and liver samples were harvested in a sterile manner for further examination. In addition, as shown in Additional file 1a, 6–8-week-old male C57BL/6J mice were given antibiotics (ABX) (vancomycin, 100 mg/kg; neomycin sulfate 200 mg/kg; metronidazole 200 mg/kg; and ampicillin 200 mg/kg) intragastrically once each day for 1 week to deplete the gut microbiota (receptor mice). Preoperatively collected feces of Sen and Res mice or low *L. murinus* patient feces and high *L. murinus* patient feces (donor mice) were resuspended in PBS at 0.125 g /ml. An amount of 0.1 ml of the solution was administered to mice in the corresponding groups orally via gastric gavage tube 1 week (6–8 mice per group). All the mice had free access to food and water, and mice were performed intestinal ischemia 60 min and reperfusion 120 min surgery after 1 week of transplantation, and then blood, ileum, kidney, lung, and liver samples were harvested in a sterile manner for further examination.

### Lactobacillus murinus pretreatment experiment

As shown in Additional file 2a, mice were randomly divided into 3 groups (6–8 mice per group): (1) Sham group; (2) I/R group; (3) I/R + *L. murinus* group. The sham group of mice were gavaged daily for 7 days with 200 μl MRS medium and then intestinal I/R was performed without SMA occlusion. The I/R group of mice were gavaged daily for 7 days with 200 μl MRS medium, and intestinal SMA was occluded for 60 min followed by 120 min reperfusion. The I/R + *L. murinus* group of mice were gavaged daily for 7 days with 200 μl 6.8 × 10^8^ CFU/ml *L. murinus* prior to establishing intestinal I/R. In addition, in a separate group of animals receiving the same protocols (20 mice per group), the survival of the mice was monitored for 5 days. To explore a single strain of *L. murinus* alleviates intestinal I/R-induced intestinal injury, GF mice were allocated randomly to 2 groups (5–6 mice per group): (1) I/R group; (2) I/R + *L. murinus* group. I/R group of GF mice, which were gavaged daily for 3 days with 200 μl MRS medium, and intestinal SMA was occluded for 60 min followed by 120 min reperfusion. I/R + *L. murinus* group of GF mice, which were gavaged daily for 3 days with 200 μl 6.8 × 10^8^ CFU/ml *L. murinus* prior to establishing intestinal I/R.

### Macrophage depletion experiment

Clodronate liposomal (Clodronate-Lipo) was administered intraperitoneally to mice 2 days before I/R to deplete macrophages. Mice were allocated randomly to 4 groups (6–8 mice per group): (1) I/R group; (2) PBS-Lipo + I/R group; (3) Clodronate-Lipo + I/R group; (4) Clodronate-Lipo + *L. murinus*+I/R group. I/R group of mice, in which intestinal SMA was occluded for 60 min followed by 120 min reperfusion, was established as described previously; PBS-Lipo + I/R group of mice, which were intraperitoneally injected with 200 μl empty liposomes (Yeasen, Shanghai, China) 48 h before establishing I/R; Clodronate-Lipo + I/R group of mice, which were intraperitoneally injected with 200 μl clodronate liposomal (Yeasen, Shanghai, China) 48 h before establishing I/R model; Clodronate-Lipo + *L. murinus* + I/R group of mice, which were given treatment of *L. murinus* and clodronate liposomal injection before establishing I/R (Additional 2b).

### Patient samples

From 2019 September to 2020 January, we recruited consecutive patients who underwent elective cardiac valve replacement or coronary artery bypass graft under cardiopulmonary bypass (CPB) at the Department of Cardiac Surgery, and healthy volunteers who underwent physical examination at the Department of Health Management, in Southern Medical University Nanfang Hospital, Guangzhou, China. Participants were not included if they (1) aged < 18 or > 75 years, (2) had chronic kidney disease, (3) had chronic digestive system diseases, previous gastrointestinal surgery, or confirmed or suspected intestinal ischemia/necrosis, (4) used antidiarrheals, laxatives or prebiotics within 1 week, or used antibiotics within 3 months. A total of 26 participants were enrolled, including 20 patients who underwent CPB and 6 healthy volunteers. There was no significant difference between the patient group and the healthy group in terms of demographic characteristics. The study protocol was approved by the Ethical Committee of Nanfang hospital, Southern Medical University (approval number NFEC-202009-k2-01).

### Fecal and blood samples

Blood samples were collected at preoperatively (T0) and at 0 h (T1), 2 h (T2), 6 h (T3), 12 h (T4), and 24 h (T5) after surgery in EDTA plasma tubes as well as in serum separator tubes for analyses of intestinal fatty acid-binding protein (IFABP) and citrulline respectively. Fecal samples were collected at preoperatively, and the relative abundance of *L. murinus* was quantified by real-time PCR. IFABP and citrulline in the plasma samples were measured at multiple time points, to allow for (T3-T0) concentration differences, respectively by means of a human IFABP ELISA Kit (Bio-Swamp, Wuhan, China) and Citrulline ELISA Kit (USCN, Wuhan, China), following the manufacturer’s instructions. The gastrointestinal complication score of the patient on the seventh day after surgery was performed according to the acute gastrointestinal injury (AGI) standard described previously [32]. The detection of *L. murinus*, IFABP, citrulline, and AGI scores were performed by researchers blinded to the group allocation.

### Microbe analysis

Feces were collected with sterilized 1.5-ml tubes before intestinal I/R and frozen at − 80 °C until DNA extraction. All extracted DNA was stored in − 20 °C until further test. The extracted fecal DNA concentration was diluted to 10 ng/μl, and quantitative real-time PCR was processed according to 16S rRNA primers, *Firmicutes* primers, and *Bacteroidetes* primers; the primers are listed in Table S1. Moreover, 16S rRNA abundance from blood was normalized to host 18S.

### 16S rRNA gene sequencing

Microbial DNA from preoperative fecal samples of mice were extracted using the classical phenol-chloroform extraction method as previously described [31, 33]. Next, the V4 region of 16S rDNA was amplified using barcode-specific primers (V4F, 5′-GTGTGYCAGCMGCCGCG GTAA-3′ and V4R, 5′-CCGGACTACNVGGG TWTCTAAT-3′), and the amplified products were quantified using QuantiFluorTM and mixed in equal amounts.

All samples were paired-end sequenced on the Illumina Hiseq PE2500 sequencing platform. Low-quality reads were filtered after quality control, and high-quality reads were assigned to operational taxonomic units (OTUs) of ≥ 97% similarity using UPARSE pipeline [34]. QIIME was applied to analyze the alpha and beta diversities, based on weighted and unweighted UniFrac distances; successively Metastasis (version 20090414) and Linear discriminant effect size (LEfSe) software [35] (version 1.0) were used to explore biomarker features in each group. The KEGG pathway analysis of the OTUs was performed using Tax4Fun [36] (version 1.0) and was performed using the OmicShare tools, a free online platform for data analysis (www.omicshare.com/tools). The calculated *p* value was gone through FDR correction, taking FDR ≤ 0.05 as a threshold.

### Peritoneal macrophage collection

Macrophages were generated from WT, TLR2^−/−^, and IL-10^−/−^ mice. Macrophages were collected as previously described [37]. Injection of 4 ml of normal saline solution into the peritoneum was used for peritoneal macrophage collection, and the mice’s abdomen was gently rubbed for 2 min to make the liquid flow in the abdominal cavity. The peritoneal fluid was sucked out and transferred into a centrifugal tube with a glue-head dropper. The amount of each suction was about 4~5 ml. The collected peritoneal lavage fluid was centrifuged at 1000 r/min for 10 min and then macrophages were cultured in Dulbecco’s modified Eagle’s medium (DMEM; SH30243.01B, Hyclone, USA) supplemented with 10% fetal calf serum, penicillin (100 U/ml, Sigma), and streptomycin (1 mg/ml) at 37 °C in a humidified incubator under 5% CO_2_/air atmosphere.

### Extraction and culture of organoids and the establishment of hypoxia-reoxygenation (H/R) models in vitro

The extraction and culture of small intestinal organoids was performed as previously described [38–40]. Briefly, after the 6–8-week-old male C57BL/6J mice were euthanized, a small intestine about 20 cm in length was taken from the end close to the stomach and placed in cold (2–8 °C) PBS to remove the membranes, blood vessels, and fat outside the intestine. The intestines are cut longitudinally and the intestines are cleaned with PBS, then cut into 2-mm pieces and transferred to the buffer. A pipette is used to continuously pipette the intestine until the supernatant is clear, then the tissue fragments are placed in 25 mL of room temperature (15–25°C) gentle cell dissociation reagent and incubated on a shaker at room temperature at 20 rpm for 15 min. The tissue fragments are resuspended in 10 mL cold (2–8° C) PBS containing 0.1% BSA and pipetted up and down three times. After standing still, the supernatant is removed and filtered with a 70-μm filter. The separated intestinal crypts were fixed onto the bottom of the dish with Matrigel (STEMCELL Technologies Inc., Shanghai, China) drops, placed in a 37 °C cell incubator, and covered with IntestiCult medium (STEMCELL Technologies Inc.) after 40 min. Organoids were cultured at 37 °C in a humidified incubator under 5% CO_2_/air atmosphere, and the growth of the organoids was observed. The small intestinal organoids began to sprout during 2–4 days of culture and formed complex bud-like structures on the 5th to 7th days. An organoid hypoxia-reoxygenation (H/R) model is established on the 5th day of organoid culture, the organoids were placed in a humid, anaerobic environment at 37 °C for 12 h, and then placed in an aerobic environment containing 5% CO_2_ in a 37 °C incubator for 4 h.

To explore *L. murinus* may promote the release of IL-10 by macrophages through TLR2 to reduce organoid H/R injury, and we established the transwell co-culture system of macrophages separately extracted from WT (WT-MΦ), TLR2^−/−^ (TLR2^−/−^-MΦ), and IL-10^−/−^ (IL-10^−/−^-MΦ) mice and intestinal organoids extracted from WT mice (WT-Org). Organoids-macrophages co-cultures were performed as previously described [41]. In our experiment, macrophages were cultured in a humidified incubator (5% CO_2_ in air) at 37 °C. 1 × 10^4^ cells were seeded in a 0.4-μm pore size polyester membrane Transwell chamber (Corning, Inc.). In total, 600 organoids were seeded per cell culture insert and maintained in IntestiCult™ medium. After 4 days, the organoid-derived monolayer containing inserts were transferred onto wells containing 500,000 macrophages and cultured for 24 h. Three hours before the establishment of the H/R model, 10% supernatant of *L. murinus* was used to stimulate the upper chamber to further confirm the *L. murinus* may require the participation of macrophages to improve the intestinal injury in vitro. The cultured cells were harvested for further imaging multicolor flow cytometry, histological staining, and RNA extraction.

### FD-4 permeability experiment

Intestinal permeability studies were performed by measurement of macromolecular transport of fluorescein isothiocyanate (FITC)-dextran 4 kD (FD-4; Sigma, Shanghai, China) [31]. One hour before the establishment of the intestinal I60/R120 model, the mice were orally administered 0.6 mg/kg FD-4 via a gastric tube, and then 100 μl plasma was harvested after intestinal I/R. The plasma concentrations of FD-4 were measured based on a reference standard curve with an excitation wavelength of 485 nm and an emission wavelength of 530 nm in a microplate fluorescence reader (SpectraMax i3x, Molecular Devices, USA).

### Gene expression analysis

A reverse transcript enzyme (TOYOBO, Tokyo, Japan) was applied to prepare cDNA according to the manufacturer’s protocol. The real-time PCR reaction was performed using the ABI Q5 real-Time PCR System with SYBR Green detection protocol (TOYOBO, Tokyo Japan). The expression of target genes in mice were normalized to house-keeping gene 18S using the 2^−^^CT^ method. The target gene primers are listed in Additional file 3.

### Protein expression and biochemical analysis

The endotoxin level was measured via commercial kit (GenScript, Nanjing, China) in Sen group and Res group, Sen feces + I/R group, and Res feces + I/R group. Plasma alanine aminotransferase (ALT) and aspartate aminotransferase (AST) were determined manually with a commercial kit (KeyGene, Nanjing, China) in Sen feces + I/R group and Res feces + I/R group.

### Histological staining

Ileum, liver, lung, and kidney samples tissue were collected and fixed in 4% paraformaldehyde. Small intestine organoid collection and fixation were performed as previously described [40]. Briefly, the medium from the well is aspirated, 1 mL of 4% paraformaldehyde is added to gently resuspend the organoids and Matrigel, the mixture is transferred to an EP tube and incubated on ice for at least 1 h. Then, it is centrifuged at 4 °C, 1500 rpm for 5 min; the paraformaldehyde is aspirated, washed with ice-cold PBS, 4 °C, 1500 rpm, and centrifuged for 5 min; the PBS is aspirated carefully, and this is repeated at least twice. Then the organoids are resuspended in cold 4% paraformaldehyde and incubated overnight at 4 °C. Then, the samples were embedded in paraffin, and 5-μm-thick sections were sliced and were stained with hematoxylin-eosin (HE) according to the experimental protocol. The degree of ileum injury after reperfusion was evaluated using modified Chiu’s method according to changes of the intestinal mucosa villus and glands [10]. Liver, lung, and kidney tissue histological damage were assessed according to previously described scoring system [42–44]. Images were captured at × 200 or × 400 with an Olympus fluorescence microscope (Olympus, Japan).

Ileum, liver, lung, and kidney tissues of mice in Sen group and Res group, Sen feces + I/R group, and Res feces + I/R group were stained with HE. Ileum of WT mice in Sham, I/R, and I/R + *L. murinus* group, low *L. murinus* feces + I/R and high *L. murinus* feces + I/R group, I/R, PBS-Lipo + I/R, Clodronate-Lipo + I/R, and Clodronate-Lipo + *L. murinus* + I/R group were stained with HE. WT-Org with NC group, H/R group, and H/R + *L. murinus* group were stained with HE, in addition, organoids from WT-Org + WT-MΦ, WT-Org + TLR2^−/−^-MΦ, and WT-Org + IL-10^−/−^-MΦ with H/R group and H/R + *L. murinus* group were stained with HE.

### Immunofluorescence

Paraffin section samples were generated as above, blocked for1 h, and incubated overnight at 4 °C with anti-ZO-1 antibody (Abcam, Cambridge, MA, USA) and anti-occludin antibody (Abcam, Cambridge, MA, USA). Then the small intestine tissue and small intestine organoids were then washed and stained with DAPI for 10 min, and Images were captured with fluorescent microscopy (Olympus, Japan). Quantification of the fluorescence intensity of ZO-1 and occludin staining of small intestine tissue were performed by automated image analysis in five randomly chosen × 200 fields of each sample, and organoid tissues were performed by automated image analysis in × 400 field of each sample.

### Flow cytometry and antibodies

The collected peritoneal lavage fluid was centrifuged at 1000 r/min for 10 min and the supernatant was removed, then further flow experiment was carried out. For intracellular cytokine staining, single-cell suspensions of macrophages were stimulated with PMA (25 ng/mL; Sigma-Aldrich, St. Louis, MO, USA) then ionomycin (1 μg/mL; Sigma-Aldrich) for 5 h at 37 °C. Brefeldin A (10 μg/mL; Sigma-Aldrich) was added after the first hour of incubation. The cells were sedimented by centrifugation at 600*g* at 4 °C and washed with MACS buffer, then nonspecific antibody binding was blocked by incubation with CD16/CD32 antibody (Cat# 14-0161-86, eBioscience, Shanghai, China) for 15 min on ice. The cell surface was stained with 0.25 μg of anti-mouse F4/80 Antigen FITC (Invitrogen eBioscience, USA), PE-Cy7 anti-mouse CD45 (BD Bioscience, USA), APC anti-mouse CD206 (Invitrogen eBioscience, USA), anti-mouse CD11C (Invitrogen eBioscience, USA), BV421 anti-mouse CD282(TLR2)(BD Bioscience, USA), and BV510 anti-mouse IL-10 (BD Bioscience, USA). F4/80^+^CD45^+^ served as a macrophage marker, with F4/80^+^CD45^+^CD206^+^CD11C^−^ cells considered to be M2 macrophages and F4/80^+^CD45^+^CD206^−^CD11C^+^ cells considered to be M1 macrophages. Flow cytometric analysis was performed on FACS Calibur (BD Biosciences, USA) instruments and analyzed using FlowJo software (Tree Star Inc.).

### Detection of organoid injury by lactate dehydrogenase (LDH) assays

The LDH kit (Nanjing Jiancheng Bioengineering Institute, Nanjing, China) was used to detect the level of LDH in the culture medium to assess organoid damage. The detection of LDH was carried out based on the manufacturers’ protocols.

### Statistical analysis

All analyses were performed using GraphPad Prism software (version 7.0). Data are presented as means ± standard error of mean (SEM). Statistical analyses were performed using two-sided Student’s *t* tests or by one-way analysis of variance (ANOVA) as indicated in the figure legends. *p* values < 0.05 were considered statistically significant.

## Results

### Characteristics of tissue injury and the gut microbiota in intestinal Sen and Res mice

**Fig. 1:**
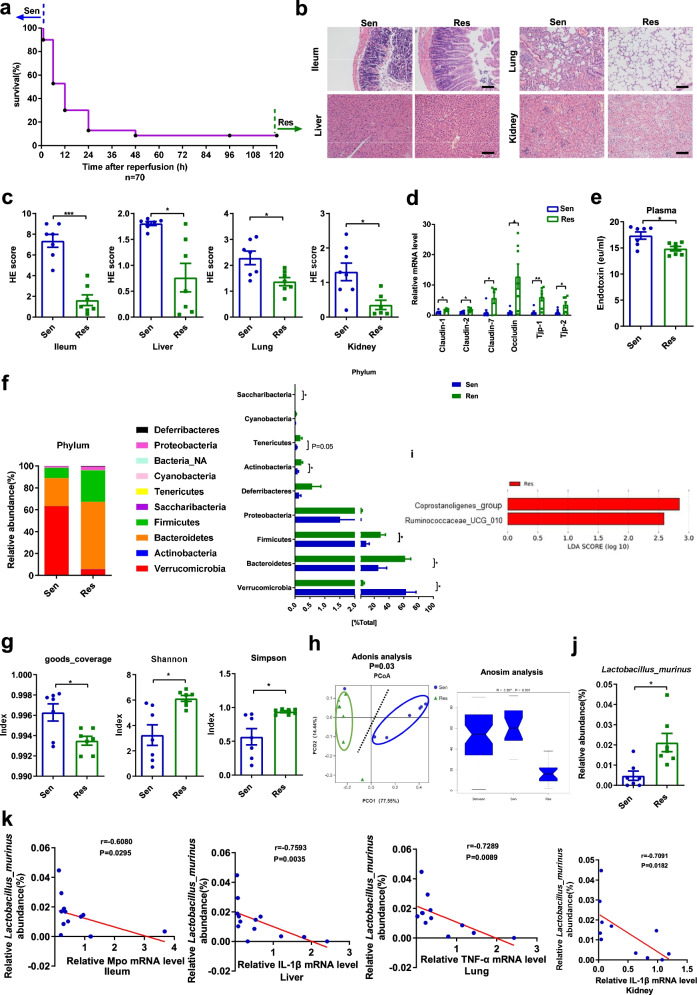
Characterization of tissue injury and the gut microbiota in Sen and Res mice. Six-to eight-week-old male C57BL/6 mice underwent intestinal ischemia for 60 min, and the survival rate was observed after reperfusion. We defined mice that died within 1 h after reperfusion as Sen mice, and those that survived for 5 days as Res mice. **a** Changes in survival rate (*n* = 70) have been shown. **b, c** HE staining of the ileum, liver, lung, and kidney and the pathology scores. Scale bar = 100 μm. **d** The mRNA levels of tight junction proteins in the ileum. **e** Relative plasma endotoxin level. **f** Relative bacterial abundance at the phyla level in the feces of mice. **g** Alpha diversity indices. **h** PCoA based on the weighted UniFrac analysis of operational taxonomic units (OTUs) and anosim analysis. **i** Histogram of the LDA score showing the enriched bacteria in the gut microbiome of the Res mice. **j** Relative abundance of *L. murinus* at the species level in the Sen and Res mice. **k** Correlation between the mRNA levels of inflammatory factors in the ileum, liver, lung, and kidney and the abundance of *L. murinus* in the Sen and Res mice. Results are expressed as the mean ± SEM. *n* = 7. ***p < 0.001, **p < 0.01, *p < 0.05 were determined by two-tailed Student’s *t* test in **c–g**, adonis analysis and anosim analysis in **h**, Spearman analysis in **k**. LDA, linear discriminant analysis; *L. murinus*, *Lactobacillus murinus*; OTUs, operational taxonomic units; PCoA, principal coordinates analysis; Sen, sensitive; Res, resistant

The survival rate of mice after intestinal I/R was observed, and it is found that mice have obvious differences in susceptibility to intestinal I/R injury. Mice that died within 1 h after reperfusion were defined as “Sen mice,” while those that survived up to 5 days were defined as “Res mice” (Fig. 1a). Compared with the Res mice, the Sen mice showed significantly aggravated histopathological injury and histology scores in the ileum, liver, lung, and kidney observed by HE staining, as well as upregulated mRNA levels of *Il-1β*, *Il-6*, and *Tnf-α.* (Fig. 1b, c and Additional file 4a). Moreover, the mRNA levels of intestinal tight junction markers in the ileum of the Res mice were markedly higher, whereas the plasma endotoxin level was reduced compared with those in Sen mice (Fig. 1d, e).

Real-time PCR and 16S DNA sequencing were used to explore whether the susceptibility or resistance of the Sen or Res mice, respectively, to intestinal I/R was related to differences in their gut microflora. There was a significant increase in the relative total bacterial load; the relative abundance of *Firmicutes* and *Bacteroidetes* as well as the *Firmicutes/Bacteroidetes* ratio in the feces of Sen mice were lower than those in the feces of Res mice (Additional file 4b, c). 16S DNA sequencing results showed that the bacterial composition in fecal samples from Sen and Res mice in terms of both bacterial phyla and class were significantly different (Fig. 1f and Additional file 4d). Alpha diversity analysis and principal coordinate analysis (PCoA) indicated that the overall structure of the gut microbiota was significantly different between the Sen and Res mice (Fig. 1g, h). To further explore the differences of gut microbial metabolic function in Sen and Res group, the 16S rRNA data were annotated with metabolic pathways from the KEGG data using Tax4Fun prediction analysis. The relative abundances of 20 metabolism-related KEGG pathways were shown in Pathway abundance heat map (Additional file 4e). In particular, the relative KEGG pathways abundances of “Metabolism; Energy Metabolism; Oxidative phosphorylation (ko00190)” and “Environmental Information Processing; Membrane Transport; Bacterial secretion system (ko03070)” were statistically upregulated in the Sen group compared with those of the Res group. LEfSe analysis indicated that the higher relative abundance of *Coprostanolignes-group* and *Ruminococcaceae-UCG-010* at the genus level in the Res mice than in the Sen mice was statistically significant (linear discriminant analysis [LDA] score > 2, Fig. 1i). Moreover, at the species level, the relative abundance of *L. murinus* was four times higher in Res than that in Sen mice, exhibiting the largest fold change (Fig. 1j). The relative abundance of *L. murinus* was negatively correlated with inflammatory factors of intestinal I/R injury (Fig. 1k).

### Gut microbiota from Res mice could independently alleviate I/R-induced tissue damage

**Fig. 2:**
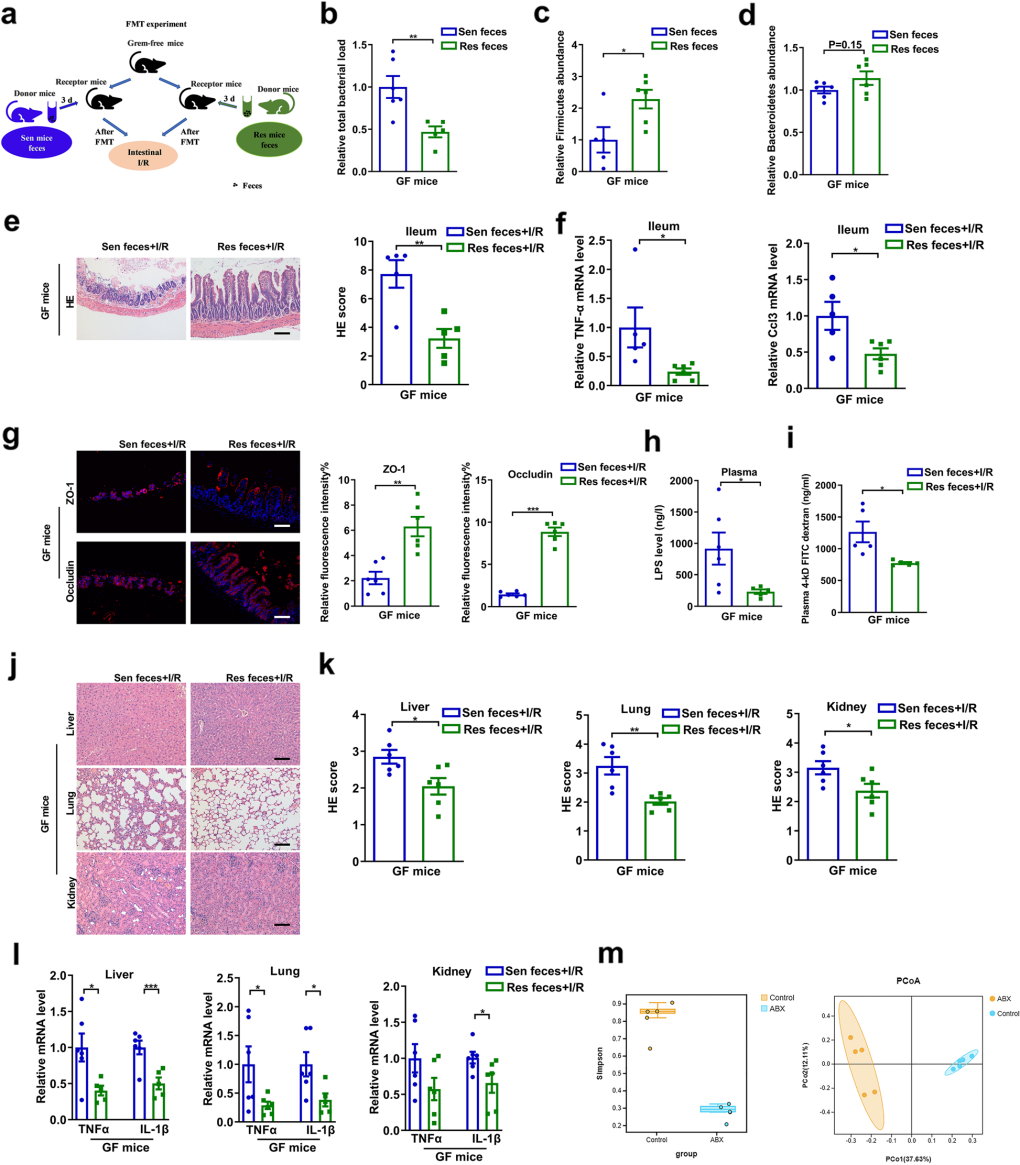
Gut microbiota from Res mice independently alleviates I/R-induced intestinal, liver, lung, and kidney tissue damage. **a** FMT experimental design. GF mice were subjected to intestinal ischemia for 60 min and reperfusion for 120 min after 3 days of transplantation. **b–d** The total bacterial load and the abundance of *Firmicutes* and *Bacteroidetes* in the feces 3 days after transplantation. **e** HE staining in the ileum. Scale bar = 100 μm. **f** The mRNA levels of proinflammatory factors in the ileum. **g–h** Tight junction mRNA levels and protein levels in the ileum and relative plasma endotoxin level. Scale bar = 100 μm. **i** FD-4 level in the plasma. **j–k** HE staining in the liver, lung, and kidney and pathology score. Scale bar = 100 μm. **l** The mRNA levels of proinflammatory factors in the liver, lung, and kidney. **m** Alpha diversity indices (Simpson) and PCoA analysis of Control and ABX-treated mice group. The results are expressed as the mean ± SEM. *n* = 5–6. ***p < 0.001, **p < 0.01, *p < 0.05 were determined using two-tailed Student’s *t* test. ABX, antibiotic; FD-4, FITC-dextran 4-KD; FMT, fecal microbiota transplantation; GF, germ-free; HE, hematoxylin-eosin; I/R, ischemia/reperfusion. PCoA, principal coordinates analysis

FMT experiments were performed to further demonstrate that the gut microbiota plays an important role in intestinal I/R injury (Fig. 2a). Recipient mouse feces were collected on the third day after FMT. GF mice that received feces from Sen mice (Sen feces group) showed a marked decrease in the levels of *Firmicutes* and an increase in the relative total bacterial load compared with mice receiving feces from Res mice (Res feces group) (Fig. 2b–d). The above results demonstrated that the FMT experiment was successful.

Intestinal I/R (60 min/120 min) was performed after 3 days of FMT. Compared with the Sen feces + I/R group, the intestinal histopathological injury and Chiu’s scores and the mRNA levels of *Tnf-α* and *Ccl3* in the ileum were significantly reduced in the Res feces + I/R group (Fig. 2e, f). As presented in Fig. 2g–i and Additional file 4f, the mRNA and protein levels of ZO-1 and occludin in the ileum were higher, while the plasma LPS level and FD-4 permeability were lower, in the Res feces + I/R group than those in the Sen feces + I/R group. Furthermore, the histopathological injury and mRNA levels of inflammatory factors in the liver, lung, and the kidney were reduced in the Res feces + I/R group compared with those in the Sen feces + I/R group (Fig. 2j–l). In addition, α and β diversity analysis showed that the diversity of intestinal microbes of ABX-treated mice was significantly lower than that of control mice and could be completely clustered (Fig. 2m). At the same time, the results of ABX-treated mice are consistent with the results of germ-free mice (Additional file 1). The FMT experiment confirmed that the gut microbiome before intestinal ischemia is closely related to the postoperative outcome of intestinal I/R.

### A single strain of L. murinus alleviates intestinal I/R-induced intestinal injury

**Fig. 3:**
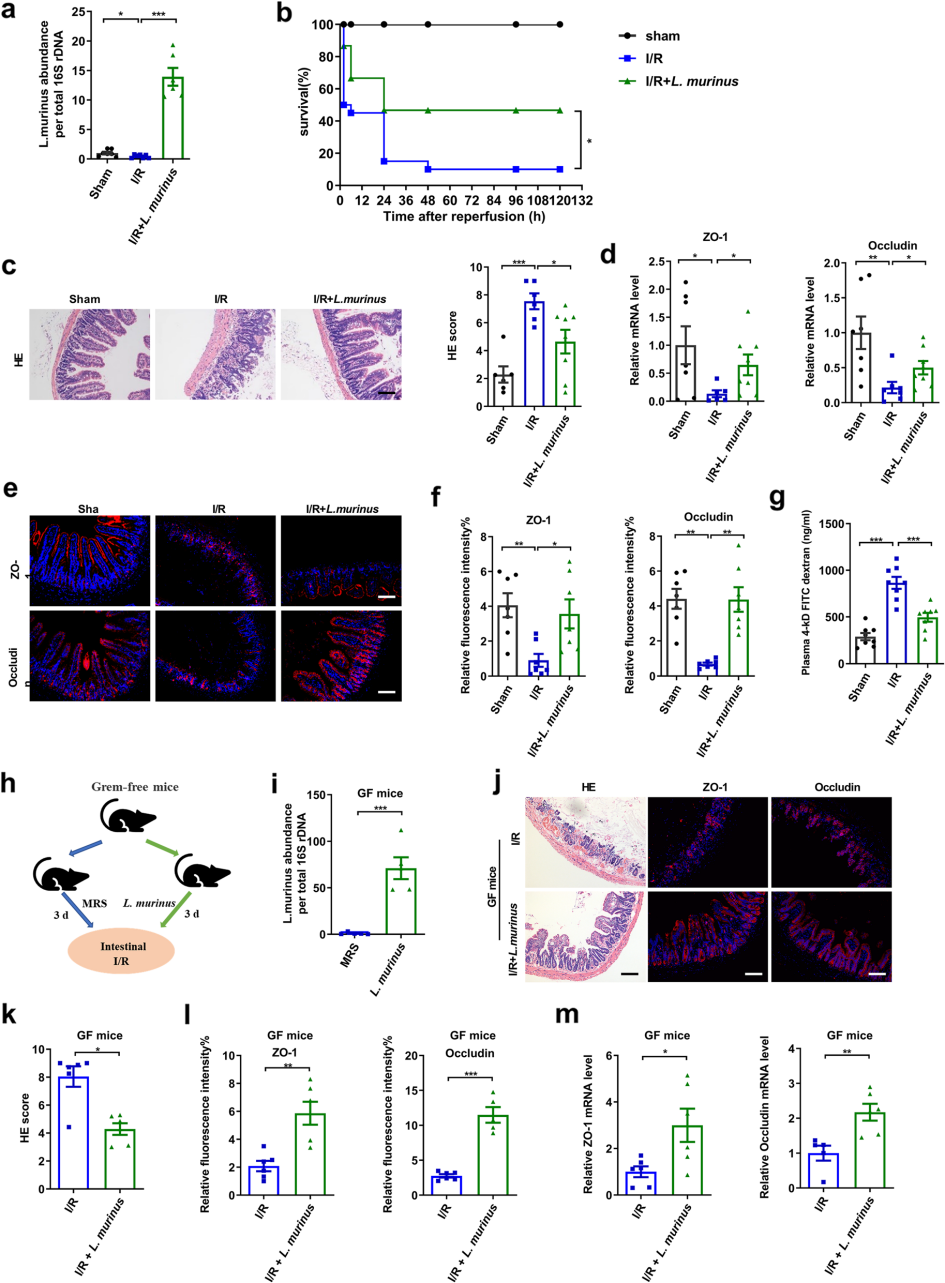
*Lactobacillus murinus* improves intestinal I/R-induced intestinal injury. **a** Relative abundance of *L. murinus* in the cecum by real-time PCR. **b** Effect of *L. murinus* on survival rate. Mice were subjected to ischemia of SMA for 60 min, and survival was monitored on day 5 after reperfusion (*n* = 20). **c** HE staining in the ileum. Representative quantification on the right. Scale bar = 100 μm (*n* = 6–8). **d–f** Relative mRNA and protein levels of ZO-1 and occludin in the ileum were measured by immunofluorescent staining. Scale bar = 100 μm (*n* = 6–8). **g** FD-4 level in the plasma (*n* = 6-8). **h** Experimental design of *L. murinus* colonized in GF mice. **i** Relative abundance of *L. murinus* in the feces (*n* = 5–6). **j–l** HE staining and ZO-1 and occludin immunofluorescent staining in the ileum. Representative quantification on the right. Scale bar = 100 μm (*n* = 5–6). **m** Tight junction mRNA levels in the ileum (*n* = 5–6). The results are expressed as the mean ± SEM. ***p < 0.001, **p < 0.01, *p < 0.05 were determined by one-way ANOVA (Tukey’s test) and log-rank test in **b**. ANOVA, analysis of variance; FD-4, FITC-dextran 4-KD; GF, grem-free; I/R, ischemia/reperfusion; *L. murinus*, *Lactobacillus murinus*; SMA, superior mesenteric artery

From the 16S rRNA gene sequencing analysis, it was found that the relative abundance of *L. murinus* was four times higher in Res mice than in the Sen mice (Fig. 1j). Moreover, the relative abundance of this species decreased significantly after intestinal I/R, as evidenced by from real-time PCR (Fig. 3a). This result prompted us to investigate whether oral administration of *L. murinus* could ameliorate intestinal I/R-induced intestinal injury. Treatment with *L. murinus* significantly increased the relative abundance of *L. murinus* in the cecum, improved survival rate, and reduced histopathological injury relative to I/R mice (Fig. 3a–c). The I/R + *L. murinus* and sham mice had markedly increased ZO-1 and occludin mRNA and protein levels, and decreased the FD-4 permeability compared with I/R mice (Fig. 3d–g). To further demonstrate the role of a single strain of *L. murinus* in intestinal I/R, GF mice were gavaged for 3 days with *L. murinus* before I/R (Fig. 3h). Real-time PCR results revealed that the relative abundance of *L. murinus* in the feces of GF mice treated with *L. murinus* was higher than in those treated with MRS medium (Fig. 3i). As presented in Fig. 3j–m, *L. murinus* reduced intestinal pathological injury, while increasing ZO-1 and occludin mRNA and protein levels in the ileum following I/R in GF mice. In addition, the results obtained for ABX-treated mice are consistent with those for GF mice (Additional file 5). The above results demonstrate that *L. murinus* alleviates intestinal I/R-induced intestinal injury in GF mice.

### Relationship between L. murinus and postoperative intestinal injury in patients undergoing cardiopulmonary bypass surgery

**Fig. 4:**
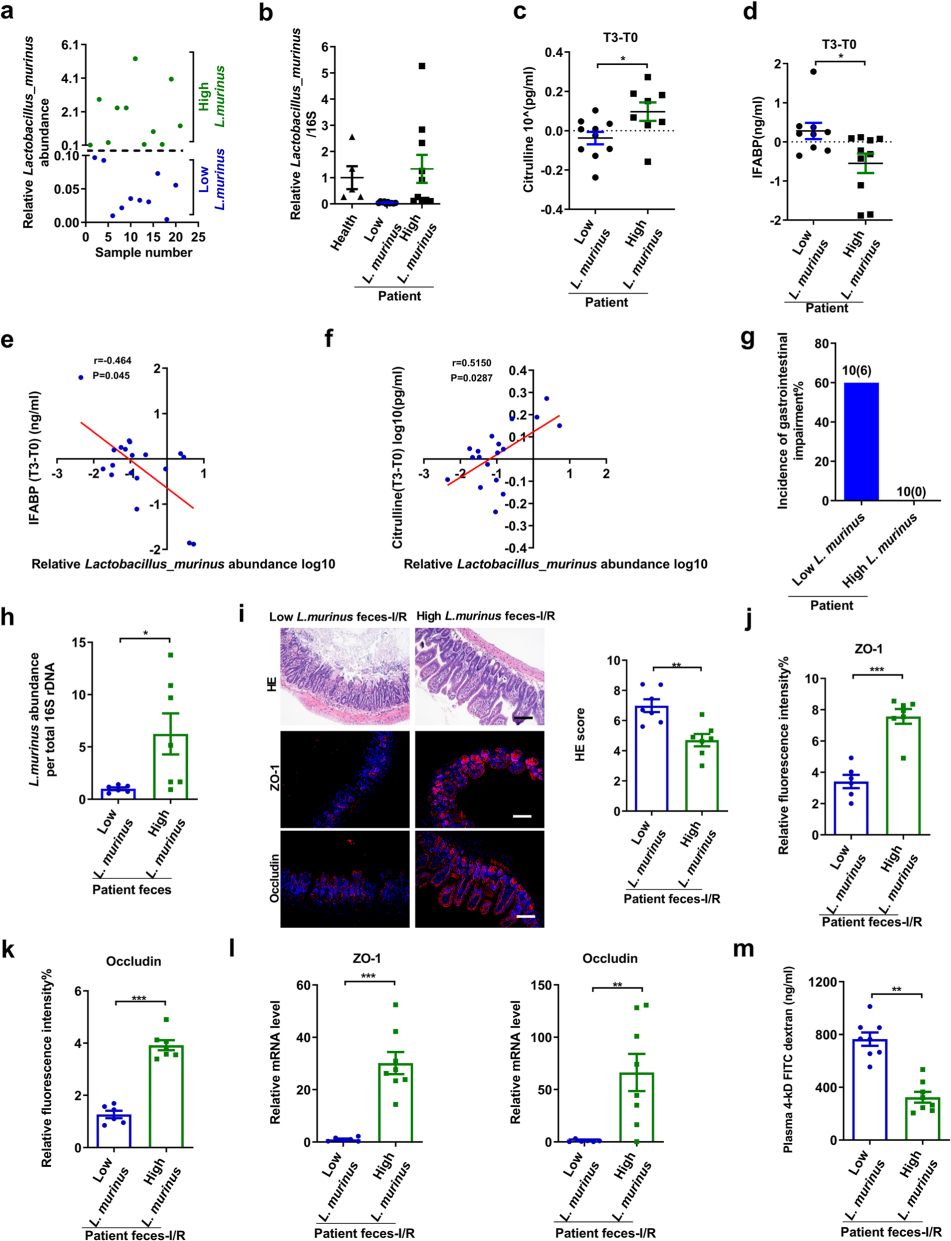
Relationship between *L. murinus* and postoperative intestinal injury in patients undergoing cardiopulmonary bypass surgery. **a, b** Relative abundance of *L. murinus* in preoperative feces of CPB patients (*n* = 20) and healthy individuals (*n* = 6). **c, d** Changes in IFABP and citrulline concentrations at 6 h postoperatively (*n* = 10). **e, f** Correlation of relative *L. murinus* abundance with serum IFABP level and citrulline level. **g** Occurrence of gastrointestinal injury in patients undergoing CPB. **h** Relative abundances of *L. murinus* in the feces by real-time PCR between low *L. murinus* feces group and high *L. murinus* feces group (*n* = 6–8). **i–k** HE staining and ZO-1 and occludin immunofluorescent staining in the ileum and representative quantification. Scale bar = 100 μm (*n* = 6–8). **l** Relative mRNA levels of ZO-1 and occludin in the ileum (*n* = 6–8). **m** FD-4 level in the plasma (*n* = 6–8). The results are expressed as the mean ± SEM. ***p < 0.001, **p < 0.01, *p < 0.05 were determined by two-tailed Student’s *t* test and Spearman analysis in **e, f**. CPB, cardiopulmonary bypass; FD-4, FITC-dextran 4-KD; IFABP, Intestinal fatty acid-binding protein; *L. murinus*, *Lactobacillus murinus*

In patients undergoing cardiac surgery, CPB is potentially responsible for intestinal ischemia (reduced blood supply and oxygen delivery) and injury; thus, it was used as a clinical model of intestinal I/R in this study [45, 46]. Firstly, the relative abundance of *L. murinus* in preoperative feces of patients undergoing CPB surgery was categorized into low (less than 0.1) and high (more than 0.1) (Fig. 4a, b). There were no differences in baseline characteristics between the two groups (Additional file 6). Plasma concentrations of citrulline and IFABP in both groups were measured as markers of absorptive enterocyte mass and intestinal failure in humans [47, 48]. There were no significant differences in the levels of citrulline and IFABP between the two groups before surgery (Additional file 7). Surprisingly, as shown in Fig. 4c–f, the abundance of *L. murinu*s in preoperative feces of patients undergoing CPB surgery inversely correlated with changes in IFABP concentration and positively correlated with changes in citrulline concentration at 6 h post-operation. Patients’ gastrointestinal function was further evaluated after 7 days post-surgery, and the occurrence rate of gastrointestinal injury in the low *L. murinus* abundance group patients was 60%. In contrast, the high *L. murinus* abundance group patients remained clinically devoid of gastrointestinal complications (Fig. 4g).

To further prove the role of *L. murinus* in ameliorating intestinal injury, an FMT experiment was conducted in which fecal bacteria preoperatively collected from high and low *L. murinus* abundance groups were separately transplanted into mice and correspondingly categorized into high *L. murinus* feces group and low *L. murinus* feces group, respectively. Real-time PCR revealed that the relative abundance of *L. murinus* in feces of the high *L. murinus* feces group was markedly higher than that in the low *L. murinus* feces group after FMT (Fig. 4h). Compared with the low *L. murinus* feces group, the high *L. murinus* feces group dramatically reduced I/R-induced intestinal pathological injury and gut permeability measured by plasma FD-4 level, and increased mRNA and protein levels of ZO-1 and occludin in the ileum (Fig. 4i–m).

### Improvement in intestinal I/R injury by L. murinus depends on macrophage participation

**Fig. 5:**
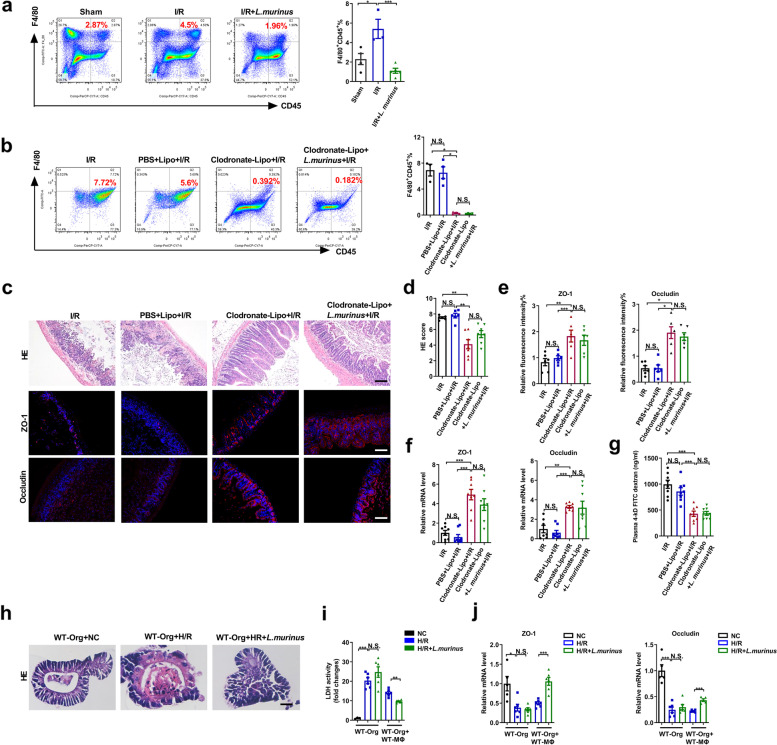
Improvement in intestinal I/R injury by *L. murinus* depends on the participation of macrophages. **a** Peritoneal macrophages were analyzed by flow cytometry for F4/80^+^CD45^+^ cells in mice in the sham group, I/R group, and IR + *L. murinus* group (*n* = 3–4). Representative quantification on the right. **b** Peritoneal macrophages were analyzed by flow cytometry for F4/80^+^ CD45^+^ cells in mice (*n* = 3–4). **c–f** HE staining and ZO-1 and occludin immunofluorescent staining in the ileum and representative quantification. Scale bar = 100 μm (*n* = 6–8). **g** FD-4 level in the plasma (*n* = 6–8). **h–j** HE staining, relative LDH levels, and tight junction mRNA levels were detected in organoids cultured alone and co-culture with macrophages after H/R. Scale bar = 20 μm (*n* = 5–6). The results are expressed as the mean ± SEM. ***p < 0.001, **p < 0.01, *p < 0.05 were determined by one-way ANOVA (Tukey’s test). ANOVA, analysis of variance; FD-4, FITC-dextran 4-KD; HE, hematoxylin-eosin; H/R, hypoxia/reoxygenation; I/R, ischemia/reperfusion; LDH, lactate dehydrogenase; *L. murinus*, *Lactobacillus murinus*

The above animal and clinical experimental results confirm that *L. murinus* has a protective effect on in intestinal I/R injury; however, the mechanism has not yet been elucidated. In a previous study, the polarization of macrophages from M1 to M2 seemingly reduced intestinal I/R injury [10].  Additionally, M1 polarization of liver macrophages may be one of the mechanisms of intestinal I/R-induced hepatic injury in mice [49]. *Lactobacillus murinus* affects the immune response; however, whether it regulates macrophages in the intestinal I/R is unclear. Flow cytometry results showed that intestinal I/R induced a significant increase in the number of macrophages (F4/80^+^CD45^+^), and this effect was reversed by *L. murinus* treatment (Fig. 5a). To further verify whether the protective effect of *L. murinus* is related to macrophages, Clodronate-Lipo was administered intraperitoneally to mice 2 days before I/R to deplete macrophages (Additional file 2b). The Clodronate-Lipo + I/R group exhibited decreased macrophage numbers (Fig. 5b) and reduced I/R-triggered histopathological injury and upregulated mRNA and protein levels of ZO-1 and occludin (Fig. 5c–f), as well as reduced the gut permeability measured by plasma FD-4 level (Fig. 5g) compared with the I/R mice and the PBS-Lipo + I/R group, but showed no statistical difference when compared with the Clodronate-Lipo + *L. murinus* + I/R group. The H/R model of small intestinal organoids was established to further confirm the results of mice. As shown in the Fig. 5h, i, *L. murinus* reduced morphological damage of intestinal organoids and LDH levels and increased the mRNA levels of ZO-1 and occludin (Fig. 5j) in the co-culture system of macrophages and organoids following H/R, but had no noticeable effect on these factors in the organoid cultured alone. Consistent with the results in vivo, the protective effect of *L. murinus* on intestinal I/R injury may depend on the participation of macrophages.

### Lactobacillus murinus may alleviate organoid H/R injury by promoting IL-10 release from macrophages via TLR2 signaling

**Fig. 6:**
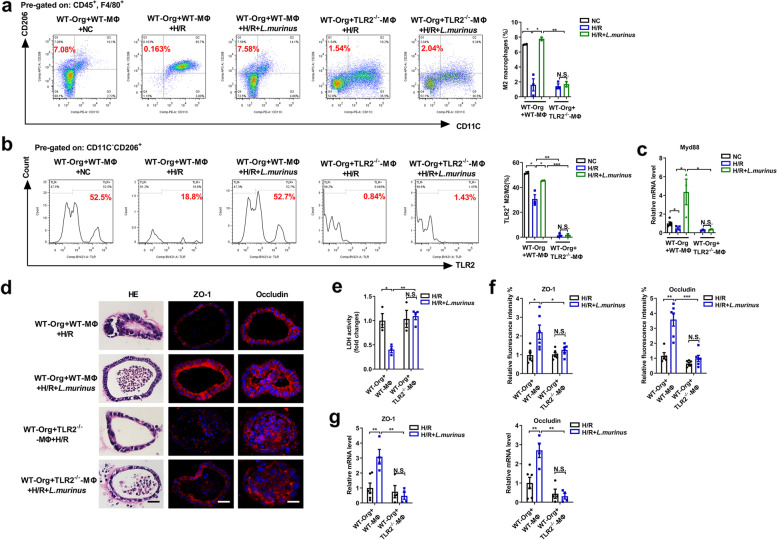
*Lactobacillus murinus* through TLR2 to reduce organoids H/R injury. **a, b** Macrophages were analyzed by flow cytometry for F4/80^+^CD206^+^CD11C^−^ cells and the expression of TLR2 on the surface M2 macrophages in the co-culture system of WT-Org and WT-MΦ or WT-Org and TLR2^−/−^-MΦ. Representative quantification on the right (*n* = 3–4). **c** Relative mRNA level of Myd88 in macrophages. **d** HE and ZO-1 and occludin immunofluorescent staining in the organoids. Scale bar = 20 μm. **e** Relative LDH levels were detected in organoids. **f, g** Relative ZO-1 and occludin fluorescence intensity and mRNA levels of ZO-1 and occludin in the organoids. The results are expressed as the mean ± SEM. *n* = 5–6. ***p < 0.001, **p < 0.01, *p < 0.05 were determined by one-way ANOVA (Tukey’s test). ANOVA, analysis of variance; H/R, hypoxia/reoxygenation; LDH, lactate dehydrogenase; *L. murinus*, *Lactobacillus murinus*; Myd88, myeloid differentiation factor 88; TLR2, toll-like receptor 2. WT-Org, organoid extracted from WT mice; WT-MΦ, macrophages extracted from WT mice; TLR2^−/−^-MΦ, macrophages extracted from TLR2^−/−^ mice

We have confirmed that the protective effect of *L. murinus* on intestinal I/R injury may depend on the participation of macrophages; hence, we explored the specific mechanism of macrophages in small intestinal organoids. TLR2 is a common receptor for the gram-positive bacterium *L. murinus*. It is unclear whether *L. murinus* reduces organoid H/R injury by activating TLR2 signaling. The transwell co-culture system of macrophages extracted from TLR2^−/−^, IL-10^−/−^, or WT mice and organoid extracted from WT mice were established. It was uncovered that *L. murinus* treatment increased the proportion of M2 macrophages, the expression of TLR2 on the surface of M2 macrophages, and the mRNA level of Myd88 following H/R in a co-culture system of WT-MΦ and WT-Org, but not in the co-culture system of TLR2^−/−^-MΦ and WT-Org (Fig. 6a–c). *Lactobacillus murinus* reduced organoids pathological injury and LDH levels, while increasing the mRNA and protein levels of ZO-1 and occludin in the co-culture system of WT-MΦ and WT-Org, but not in the co-culture system of TLR2^−/−^-MΦ and WT-Org (Fig. 6d–g).

**Fig. 7:**
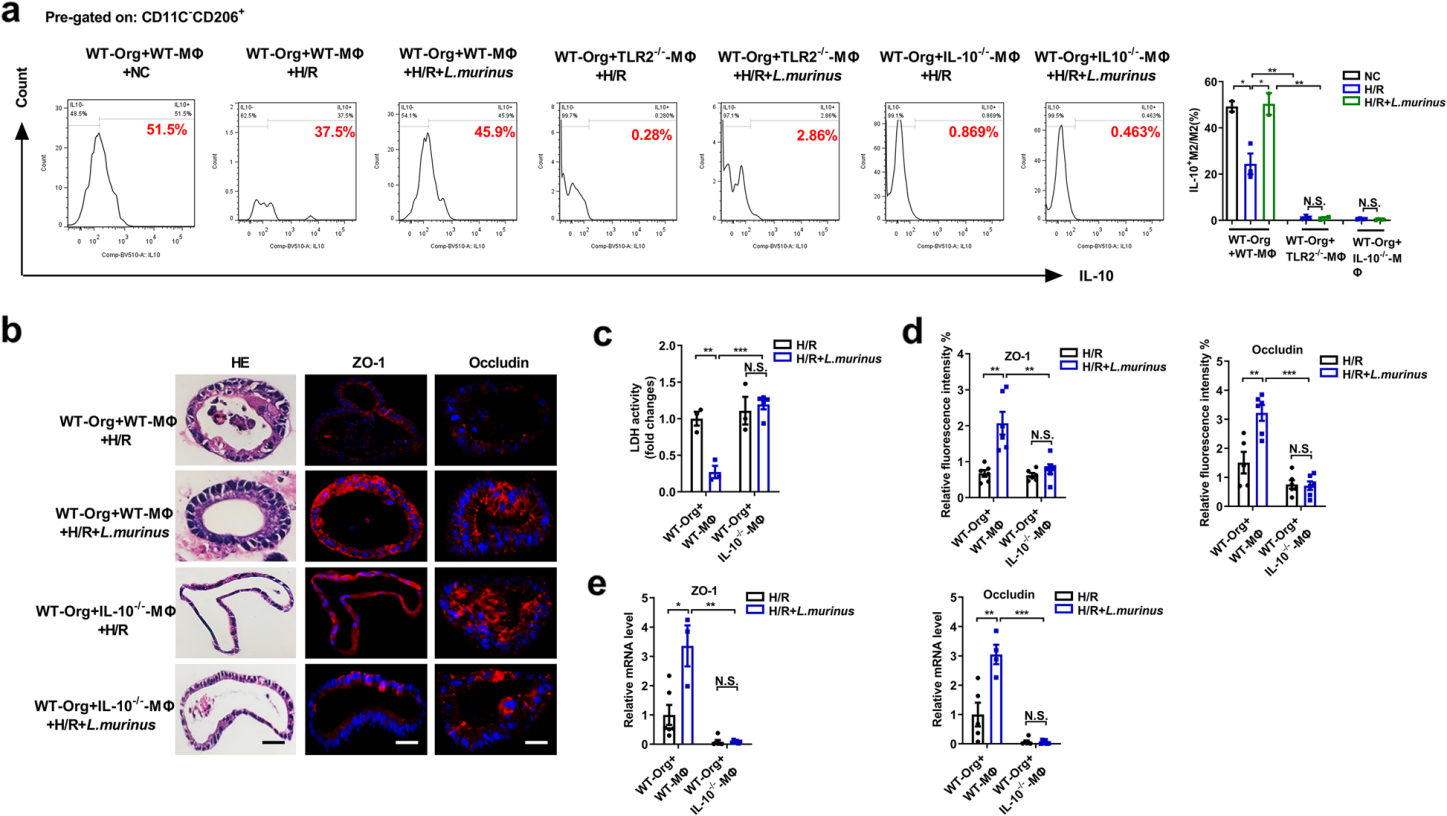
*Lactobacillus murinus* may alleviate organoids H/R injury by promoting IL-10 release from macrophages via TLR2 signaling. **a** The proportion of IL-10^+^M2 macrophages/total M2 macrophages were analyzed by flow cytometry in the co-culture system of WT-Org and WT-MΦ or WT-Org and TLR2^−/−^-MΦ or WT-Org and IL-10^−/−^-MΦ. Representative quantification on the right (*n* = 3–4). **b** HE and ZO-1 and occludin immunofluorescent staining in the organoids. Scale bar = 20 μm. **c** Relative LDH levels were detected in organoids. **d, e** Relative ZO-1 and occludin fluorescence intensity and mRNA levels of ZO-1 and occludin in the organoids. The results are expressed as the mean ± SEM. *n* = 5–6. ***p < 0.001, **p < 0.01, *p < 0.05 were determined by one-way ANOVA (Tukey’s test). ANOVA, analysis of variance; H/R, hypoxia/reoxygenation; IL-10, interleukin-10; LDH, lactate dehydrogenase; *L. murinus*, *Lactobacillus murinus*; TLR2, toll-like receptor 2. WT-Org, organoid extracted from WT mice; WT-MΦ, macrophages extracted from WT mice; TLR2^−/−^-MΦ, macrophages extracted from TLR2^−/−^ mice; IL-10^−/−^-MΦ, macrophages extracted from IL-10^/−^ mice

In addition, flow cytometry results showed that *L. murinus* suppressed the decrease in the proportion of IL-10^+^ M2 macrophages/total M2 macrophages induced by H/R in the transwell co-culture system of WT-MΦ and WT-Org, but not in the transwell co-culture system of TLR2^−/−^-MΦ and WT-Org, IL-10^−/−^-MΦ and WT-Org (Fig. 7a). The co-culture system of IL-10^−/−^-MΦ and WT-Org was established to explore the role of IL-10 released by macrophages in the protective mechanism of *L. murinus* on organoid H/R pathological injury. *Lactobacillus murinus* reduced organoid pathological injury and LDH levels, while increasing the mRNA and protein levels of ZO-1 and occludin in the co-culture system of WT-MΦ and WT-Org following H/R, but not in that of IL-10^−/−^-MΦ and WT-Org co-culture system (Fig. 7b–e). The above results indicated that *L. murinus* may promote the release of IL-10 from M2 macrophages through TLR2 signaling to alleviate organoid H/R injury in the co-culture system of macrophages and organoids.

## Discussion

In the present study, for the first time, we confirmed that mice with the same genetic background exhibited vast differences in sensitivity to intestinal I/R injury, owing to, at least partly, differences in the fecal microbiome of mice before intestinal ischemia. Furthermore, our results revealed that the relative abundance of *L. murinus* at the species level in the feces from Res mice before intestinal ischemia was significantly higher than that in Sen mice and was negatively correlated with the levels of inflammatory factors following intestinal I/R. Clinical evidence also showed that the abundance of *L. murinus* in preoperative feces of patients undergoing CPB surgery was closely related to the degree of intestinal I/R injury after surgery. FMT results indicated that the abundance of *L. murinus* in preoperative feces of patients is related to different sensitivities of mice to intestinal I/R injury. Altered Schaedler flora contains *L.murinus*-colonized mice reduced leukocyte deposition in mesenteric I/R injury as compared to conventionally raised specific pathogen-free controls mice [9]. More importantly, treatment with *L. murinus* significantly prevented intestinal I/R-induced intestinal injury and improved mouse survival, which may depend on macrophage involvement. Moreover, in vitro experiments indicated that promoting the release of IL-10 from macrophages through TLR2 may be a potential mechanism for *L. murinus* to ameliorate intestinal I/R injury, thus shedding light on a novel mechanism of intestinal I/R injury and new therapeutic strategy for clinical practice.

It is well known that patients suffering from shock or undergoing some surgical procedures, including CPB, have different outcomes after receiving medical treatment. Nevertheless, the reason for the difference remains unclear. In this study, we employed a murine model of intestinal I/R and identified that mice with the same genetic background significantly differ in terms of sensitivity to intestinal I/R injury. Especially, 16S DNA sequencing and FMT results showed that the gut microbiome is involved in the postoperative outcome of intestinal I/R.

Gut microbiota is a complex ecosystem susceptible to the surrounding environment and diet [50]. We have previously confirmed that intestinal I/R induces significant intestinal flora disorders and indicated that intestinal microbial metabolites play an important regulatory role in intestinal I/R injury [40, 51]. Previous antibiotic experiments have shown that depletion of gut commensal bacteria can attenuate intestinal I/R injury [52]. However, the extensive diversity of the gut microbiome hinders the precise determination of whether specific microbes are associated with I/R development or progression. For the first time, herein, we found that the abundance of *L. murinus* was significantly higher in the Res mice than that in the Sen mice. *Lactobacillus murinus* strains have previously been isolated and identified from rats, mice, porcine and canine species, and humans [53, 54]. Few reports have indicated the application of *L. murinus* in host health and disease. It has been shown that *L. murinus* may be used as a potential probiotic to reduce the incidence of delayed sepsis in neonates [55]. In addition, another study showed that *L. murinus* HU-1 improved the abnormal neural behavior of offspring mice caused by maternal dysregulation [56]. Further, treatment of mice with *L. murinus* prevented salt-sensitive hypertension by modulating T helper 17 (Th17) cells [30]. Furthermore, *L. murinus* improved colitis by inducing Treg cell differentiation in colitis model [7]. Various *L. murinus* strains have been further characterized as potential probiotics in the food formulation industry, and immune cells such as Th cells and Treg cells play an important role in the treatment of various diseases by *L. murinus*. With increased public interest in *L. murinus*-containing probiotics, the impacts of *L. murinus* on intestinal I/R injury are beginning to be unraveled.

At present, macrophages are considered a potential therapeutic intervention target for inflammatory diseases and cancer [57]. Macrophages exert a protective role for the gut barrier under steady-state conditions [58], in addition, macrophages have a high level of plasticity and can adapt their phenotype in response to environmental stimuli. Studies have shown that the increased susceptibility to colitis after liposome treatment was related to the depletion of resident macrophages in the colon [59]. Furthermore, a previous study has demonstrated that depletion of intestinal resident macrophages prevents I/R injury in the gut [60]. In our study, clodronate-liposome eliminated macrophages and exerted a certain protective effect on the intestinal morphological damage in the intestinal I/R mouse model, consistent with the results reported in the literature [60]. In addition, *L. murinus* reduced morphological damage of intestinal organoids and LDH levels in the co-culture system of macrophages and small intestinal organoids following H/R, but had no obvious effect on these factors in the organoids cultured alone; thus, the protective effect of *L. murinus* on organoid H/R injury depends on the participation of macrophages. Three-dimensional intestinal organoid culture was first established in 2009 [61]. Compared with simple intestinal epithelial cell lines, organoids have the physiology of natural intestinal epithelium and functional diversity [39, 62, 63]. Compared with in vivo experiments, organoids systems in vitro avoided the interference of multiple complex factors in vivo and thus are more convenient and accurate. Here, we established an intestinal organoids H/R model in vitro to simulate intestinal I/R injury and confirmed that it is reliable for studying the mechanism and treatment of intestinal I/R injury.

The immune system recognizes microorganisms through pattern recognition receptors. Dectin-1 is a C-type lectin receptor (CLR) expressed in dendritic cells and macrophages. Inhibition of dectin-1 signaling ameliorates colitis by inducing *L. murinus*-mediated regulatory T cell expansion in the intestine [7]. The microbiota protects against I/R-induced intestinal injury through nucleotide-binding oligomerization domain-containing protein 2 (NOD2) signaling [64]. Gut microbiota restricts NETosis in acute mesenteric I/R injury [65]. TLR activation by commensal bacteria has an important impact on intestinal epithelial homeostasis. Previous literature indicates that the presence of commensal bacterial TLR signaling might improve neonatal murine I/R intestinal injury [66]. The TLR2 receptor complexes play a key mechanistic role in *L. acidophilus*-mediated protection against dextran sodium sulfate-induced intestinal epithelial tight junction barrier destruction [67]. In our study, *L. murinus* reduced organoid pathological injury in the co-culture system of WT-MΦ and WT-Org, but not in the co-culture system of TLR2^−/−^-MΦ and WT-Org. All TLRs, except TLR3, use a common adaptor protein, MyD88, to transduce activation signals. It has been confirmed that the MyD88 signaling pathway may inhibit I/R injury in the small intestine by inducing COX-2 expression [68]. It has been confirmed that inhibition of the TLR4 signaling may reduce intestinal I/R injury [69, 70], lung injury induced by intestinal I/R [71, 72], and neuroinflammation and cognitive dysfunction caused by intestinal I/R [73]. TLR5 deficiency prevented lung inflammatory responses and vascular permeability after intestinal I/R injury [74].

Nevertheless, the reported role of TLR2/Myd88 signaling in intestinal I/R injury is controversial. Compared with WT mice, TLR2^−/−^ mice have a dysregulated mucosal innate immune response and fail to produce a protective response after intestinal I/R [75]. However, some studies reported that TLR2^−/−^ mice have less intestinal damage and inflammation than WT mice [76, 77]. In addition, Liang et al. revealed that bifidobacterial and lactobacilli induced IL-10 secretion in macrophages by activating TLR2 and MyD88 pathways, conferring a protection in hosts suffering from inflammatory diseases [78]. Another group observed TLR2-mediated secretion of IL-10 and immune suppression in response to phagosome-confined *Listeria monocytogenes* [79]. Studies have shown that the effect of *L. plantarum* on IL-10 production in mouse peritoneal macrophages was mediated by TLR2-dependent ERK activation [80]. Currently, the role of IL-10 in intestinal I/R is currently controversial. IL-10 can prevent systemic and local acute inflammation after intestinal I/R [25]. Conversely, it has been reported that IL-10 does not protect from intestinal I/R injury [26, 27]. In our study, the experiments in vitro indicated that promoting the release of IL-10 from macrophages through TLR2 may be a potential mechanism for *L. murinus* to ameliorate intestinal I/R injury, thus shedding light on a novel mechanism of intestinal I/R injury.

Although differences in the gut microbiota at the individual level may lead to differences in the severity of intestinal I/R injury in patients, the gut microbiome is a complex system where different strains may act together to affect the intestine. Therefore, there is no clear evidence that one individual strain is the most beneficial. However, our study has some limitations; this study focused on the effect of *L. murinus* on TLR2, and other pattern recognition receptors were not assessed. In addition, TLR2-flox CX3CR1-Cre mice were not used in this study to generate tissue-specific *Tlr2* knockout in macrophages. Although the co-culture system established by organoids extracted from control mice and macrophages extracted from TLR2^−/−^ mice, and the co-culture system established by organoids extracted from control mice and macrophages extracted from IL-10^−/−^ mice in vitro has confirmed the role of TLR2 on the surface of macrophages, and IL-10 from macrophages in reducing the H/R injury of organoids by *L. murinus*, this does not rule out that other immune cells that express TLR2 and release IL-10 also play the same or similar roles in vivo. In addition, there is no conditional knockout of TLR2 and IL-10 of macrophages to prove the role of TLR2 on the surface of macrophages and IL-10 released from macrophages in reducing intestinal I/R injury by *L. murinus*. Therefore, promoting the release of IL-10 from macrophages through TLR2 may be a potential mechanism for *L. murinus* to ameliorate intestinal I/R injury, but our experimental results may not confirm that the TLR2-IL-10 signaling in macrophages is the only important pathway for *L. murinus* to reduce intestinal I/R injury. The potential role of other immune cells that express TLR2 and release IL-10 in improving intestinal I/R injury by *L. murinus* which cannot be completely ruled out is a major limitation of this study. In addition, our pilot study on patients was conducted on a limited number of individuals and needs to be validated in a larger cohort. In the future, metagenomic and metabolomic analyses and clinical studies should be performed to confirm the involvement of *L. murinus* in intestinal I/R injury and develop effective probiotics, and the role of other pattern recognition receptors should be further explored.

## Conclusions

Taken together, our results suggest that the gut microbiome is involved in the postoperative outcome of intestinal I/R. *Lactobacillus murinus* alleviates intestinal I/R injury through macrophages, and promoting the release of IL-10 from macrophages through TLR2 may be a potential mechanism for *L. murinus* to ameliorate organoid H/R injury in vitro. This study sheds light on a novel mechanism of intestinal I/R injury and suggests that the therapy via targeting the microbiome is a promising strategy to prevent intestinal I/R injury.

## Supplementary Information

**Additional file 1: Figure S1.** Gut microbiota from Res mice independently alleviates I/R-induced intestinal, liver, lung, and kidney tissue damage. **a** FMT experimental design. Antibiotic-treated mice were subjected to intestinal ischemia for 60 min and reperfusion for 120 min after 1 week of transplantation. **b** The total bacterial load and the abundance of *Firmicutes* in the feces 1 week after transplantation. **c** HE staining in the ileum and pathology scores. Scale bar=100 μm. **d** Relative plasma endotoxin level. **e** The mRNA levels of proinflammatory factors in the ileum. **f, g** Tight junction mRNA levels and protein levels in the ileum and representative quantification. Scale bar=100 μm. **h** HE staining in the liver, lung and kidney and the pathology scores. Scale bar=100 μm. **i** The mRNA levels of proinflammatory factors in the liver, lung and kidney. The results are expressed as the mean ± SEM. *n* = 6-8. ***p<0.001, **p<0.01, *p<0.05 were determined using two-tailed Student’s t-test. FMT, fecal microbiota transplantation; HE, hematoxylin-eosin; I/R, ischemia/reperfusion.

**Additional file 2: Figure S2.** Experimental design. **a** *L. murinus* pretreatment experiment design. **b** Macrophage depletion experiment design.

**Additional file 3: Table S1.** Primers sequence.

**Additional file 4: Figure S3.** Characterization of tissue injury and the gut microbiota in the Sen and Res mice. **a** The mRNA levels of proinflammatory factors in the ileum, liver, lung and kidney. **b, c** The total bacterial load and the level of *Firmicutes* and *Bacteroidetes* as well as the *Firmicutes*/*Bacteroidetes* ratio in the feces. **d** Relative bacteria abundance at the class level in the feces. **e** Heatmap of the expression values of 20 signaling pathways in each sample. **f**. Relative mRNA levels of ZO-1 and occludin in the ileum **g** Relative levels of ALT and AST in the plasma of GF mice. The results are expressed as the mean ± SEM. *n* = 6-8. ***p<0.001, **p<0.01, *p<0.05 were determined by two-tailed Student’s t-test. GF, germ-free; Sen, sensitive; Res, resistant.

**Additional file 5: Figure S4.** *Lactobacillus murinus* improves intestinal I/R-induced intestinal injury in antibiotic-treated mice. **a.** Experimental design. The WT mice were randomly divided into the following groups. Group ABX + I/R, in which ABX was administered 1w before intestinal ischemia; Group ABX + I/R + *L. murinus*, treatment of mice with ABX for a week, and then mice were gavaged daily for 7 days with *L. murinus*. **b** Relative abundance of *L. murinus* in the cecum after ABX clearance experiment. **c-e** HE staining and ZO-1 and occludin immunofluorescent staining in the ileum. Representative quantification on the right. Scale bar=100 μm. f Tight junction mRNA levels in the ileum. g FD-4 level in the plasma. The results are expressed as the mean ± SEM. *n* = 8. ***p<0.001, **p<0.01, *p<0.05 were determined by two-tailed Student’s t-test. ABX, antibiotic; FD-4, FITC Dextran 4-KD; I/R, ischemia/reperfusion; HE, hematoxylin-eosin; *L. murinus*, Lactobacillus murinus; SMA, superior mesenteric artery.

**Additional file 6: Table S2.** Characteristics of patients with cardiopulmonary bypass.

**Additional file 7: Figure S5.** The level of IFABP and citrulline in serum before surgery between low *L. murinus* abundance group and high *L. murinus* abundance group.

**Additional file 8.** Figure abstract.

## Data Availability

The raw sequencing data generated in this study have been deposited in NCBI Sequence Read Archive (http://www.ncbi.nim.nih.gov/sra) under the accession numbers PRJNA661144 and PRJNA763009. All other data associated with this study are present in the paper or Supplementary Materials.

## Abbreviations

ABX: Antibiotic
AGI: Acute gastrointestinal injury
ANOVA: Analysis of variance
ALT: Alanine aminotransferase
AST: Aspartate aminotransferase
Clodronate-Lipo: Clodronate liposomal
CLR: C-type lectin receptor
CPB: Cardiopulmonary bypass
Cre: Cyclization recombinase
DMEM: Dulbecco’s modified Eagle’s medium
FD-4: FITC-dextran 4
FMT: Fecal microbiota transplantation
GF: Germ-free
HE: Hematoxylin-eosin
H/R: Hypoxia/reoxygenation
IFABP: Intestinal fatty acid-binding protein
IL-10: Interleukin-10
I/R: Ischemia/reperfusion
LDA: Linear discriminant analysis
LDH: Lactate dehydrogenase
LEfSe: Linear discriminant effect size
*L. murinus*: *Lactobacillus murinus*
MΦ: Macrophages
MRS: Man Rogosa Sharpe
Myd88: Myeloid differentiation factor 88
NOD2: Nucleotide-binding oligomerization domain-containing protein 2
Org: Organoid
OTUs: Operational taxonomic units
PBS: Phosphate-buffered saline
PCoA: Principal coordinates analysis
PCR: Polymerase chain reaction
rmIL-10: Recombinant murine interleukin-10
SEM: Standard error of mean
Sen: Sensitive
SMA: Superior mesenteric artery
TLR2: Toll-like receptor 2
Res: Resistant
WT: Wild-type
