# Supplementary figures and images for: Lactobacillus murinus alleviate intestinal ischemia/reperfusion injury through promoting the release of interleukin-10 from M2 macrophages via Toll-like receptor 2 signaling
Source: Microbiome. 2022 Mar 3;10:38. doi: 10.1186/s40168-022-01227-w (PMC8896269; doi:10.1186/s40168-022-01227-w)

Figure S1

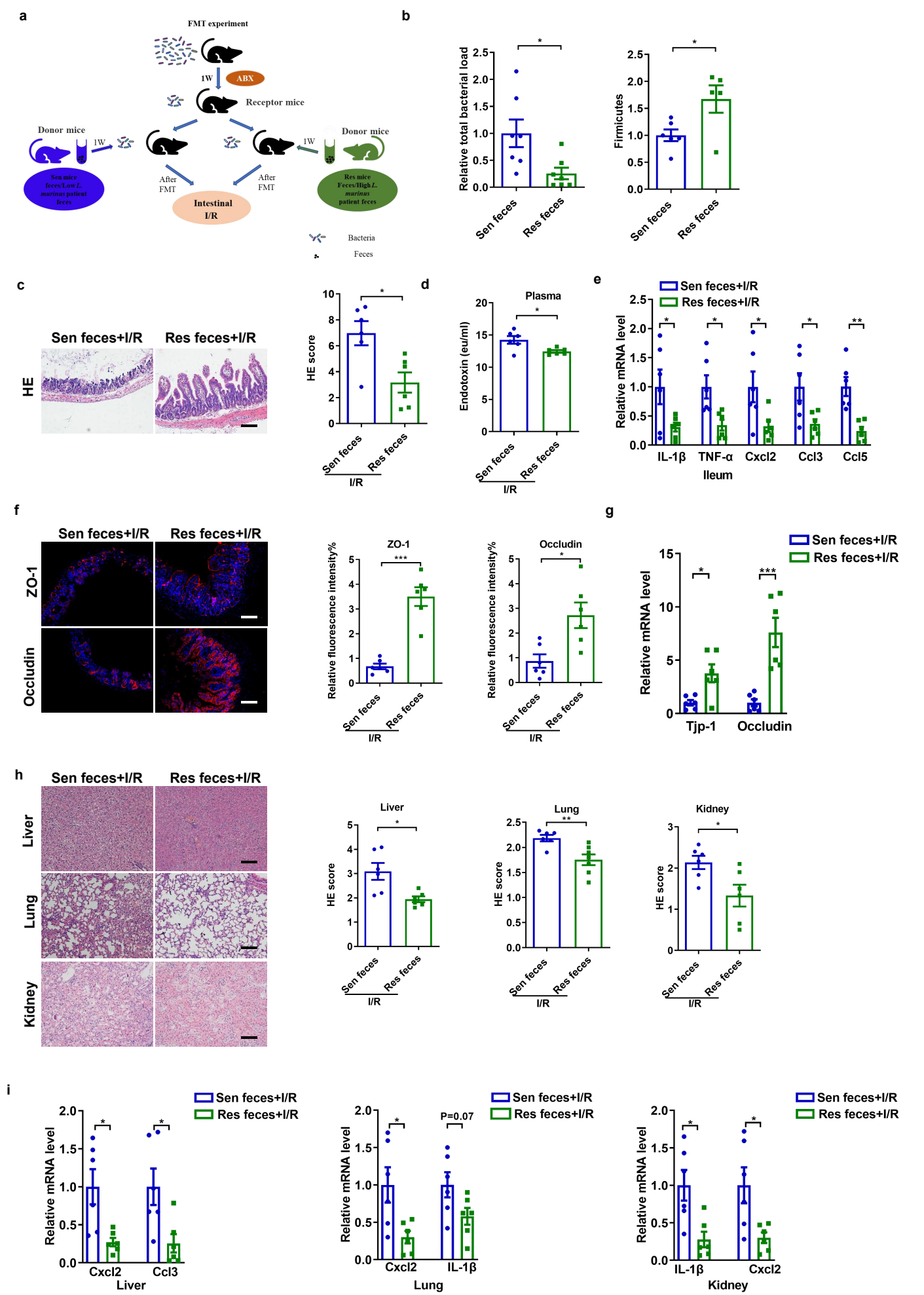

Supplement: Supplementary file 2 — Additional file 1: Figure S1. Gut microbiota from Res mice independently alleviates I/R-induced intestinal, liver, lung, and kidney tissue damage. a FMT experimental design. Antibiotic-treated mice were subjected to intestinal ischemia for 60 min and reperfusion for 120 min after 1 week of transplantation. b The total bacterial load and the abundance of Firmicutes in the feces 1 week after transplantation. c HE staining in the ileum and pathology scores. Scale bar=100 μm. d Relative plasma endotoxin level. e The mRNA levels of proinflammatory factors in the ileum. f, g Tight junction mRNA levels and protein levels in the ileum and representative quantification. Scale bar=100 μm. h HE staining in the liver, lung and kidney and the pathology scores. Scale bar=100 μm. i The mRNA levels of proinflammatory factors in the liver, lung and kidney. The results are expressed as the mean ± SEM. n = 6-8. ***p<0.001, **p<0.01, *p<0.05 were determined using two-tailed Student’s t-test. FMT, fecal microbiota transplantation; HE, hematoxylin-eosin; I/R, ischemia/reperfusion. [file 40168_2022_1227_MOESM2_ESM.pdf]

Figure S2

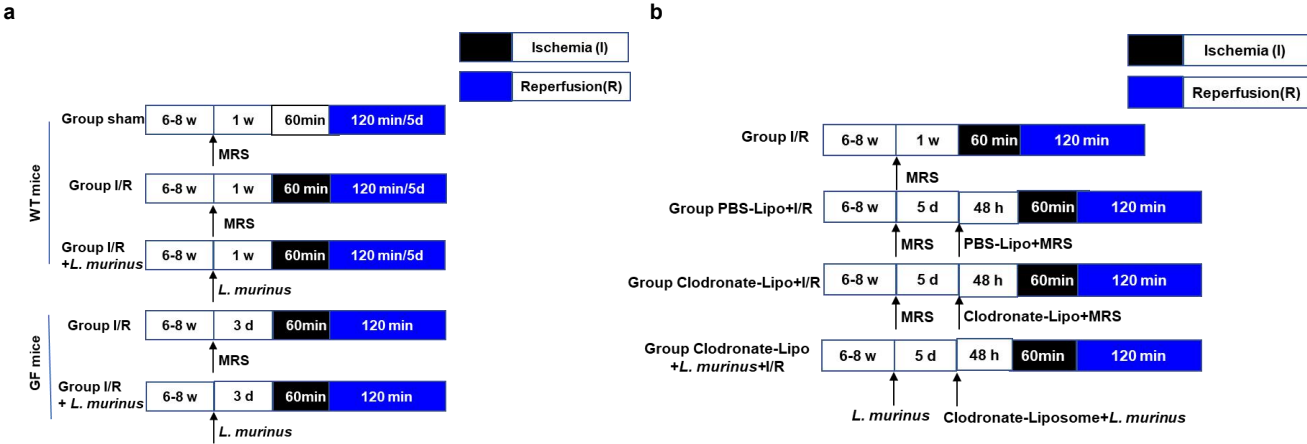

Supplement: Supplementary file 3 — Additional file 2: Figure S2. Experimental design. a L. murinus pretreatment experiment design. b Macrophage depletion experiment design. [file 40168_2022_1227_MOESM3_ESM.pdf]

Figure S3

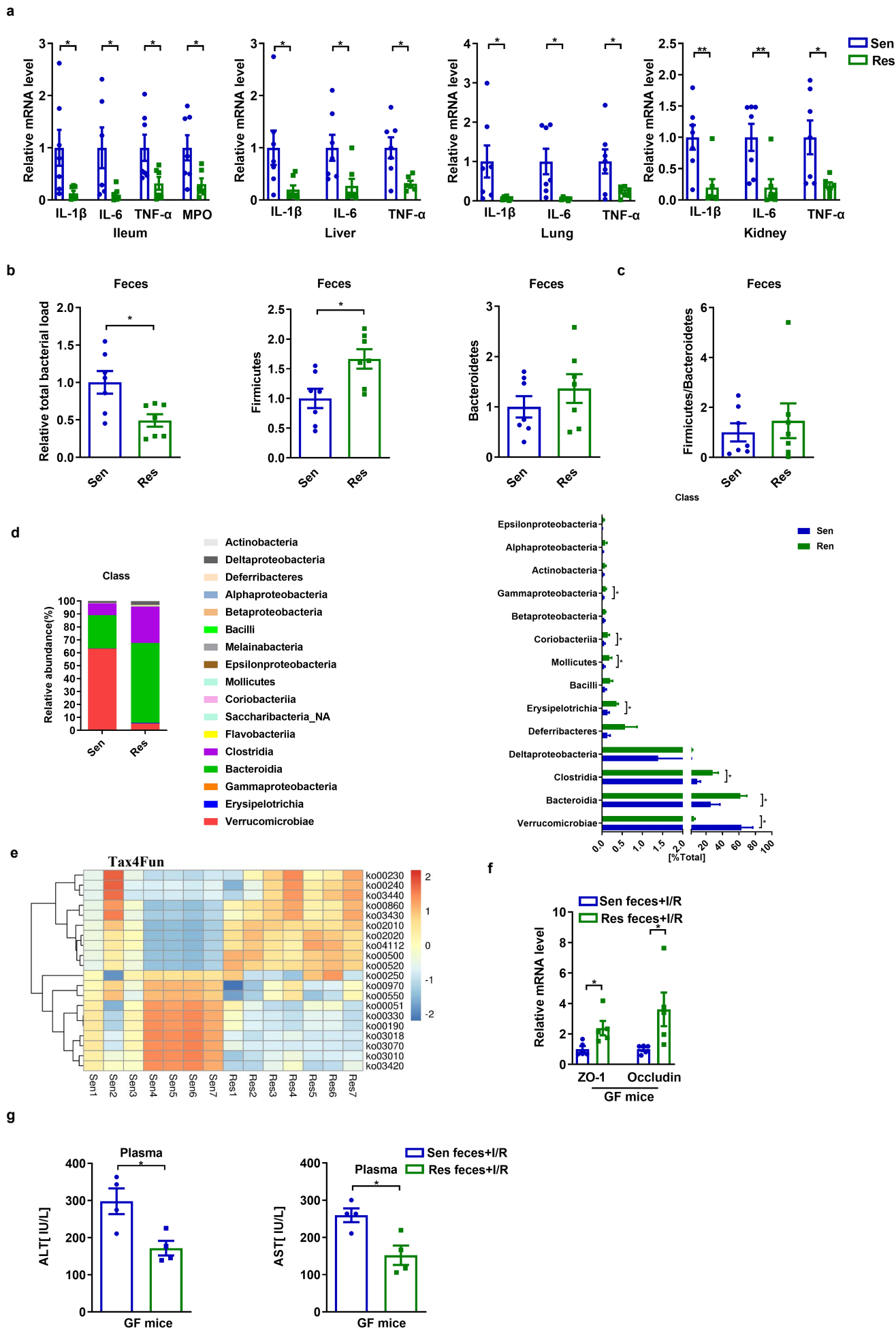

Supplement: Supplementary file 5 — Additional file 4: Figure S3. Characterization of tissue injury and the gut microbiota in the Sen and Res mice. a The mRNA levels of proinflammatory factors in the ileum, liver, lung and kidney. b, c The total bacterial load and the level of Firmicutes and Bacteroidetes as well as the Firmicutes/Bacteroidetes ratio in the feces. d Relative bacteria abundance at the class level in the feces. e Heatmap of the expression values of 20 signaling pathways in each sample. f. Relative mRNA levels of ZO-1 and occludin in the ileum g Relative levels of ALT and AST in the plasma of GF mice. The results are expressed as the mean ± SEM. n = 6-8. ***p<0.001, **p<0.01, *p<0.05 were determined by two-tailed Student’s t-test. GF, germ-free; Sen, sensitive; Res, resistant. [file 40168_2022_1227_MOESM5_ESM.pdf]

Figure S4

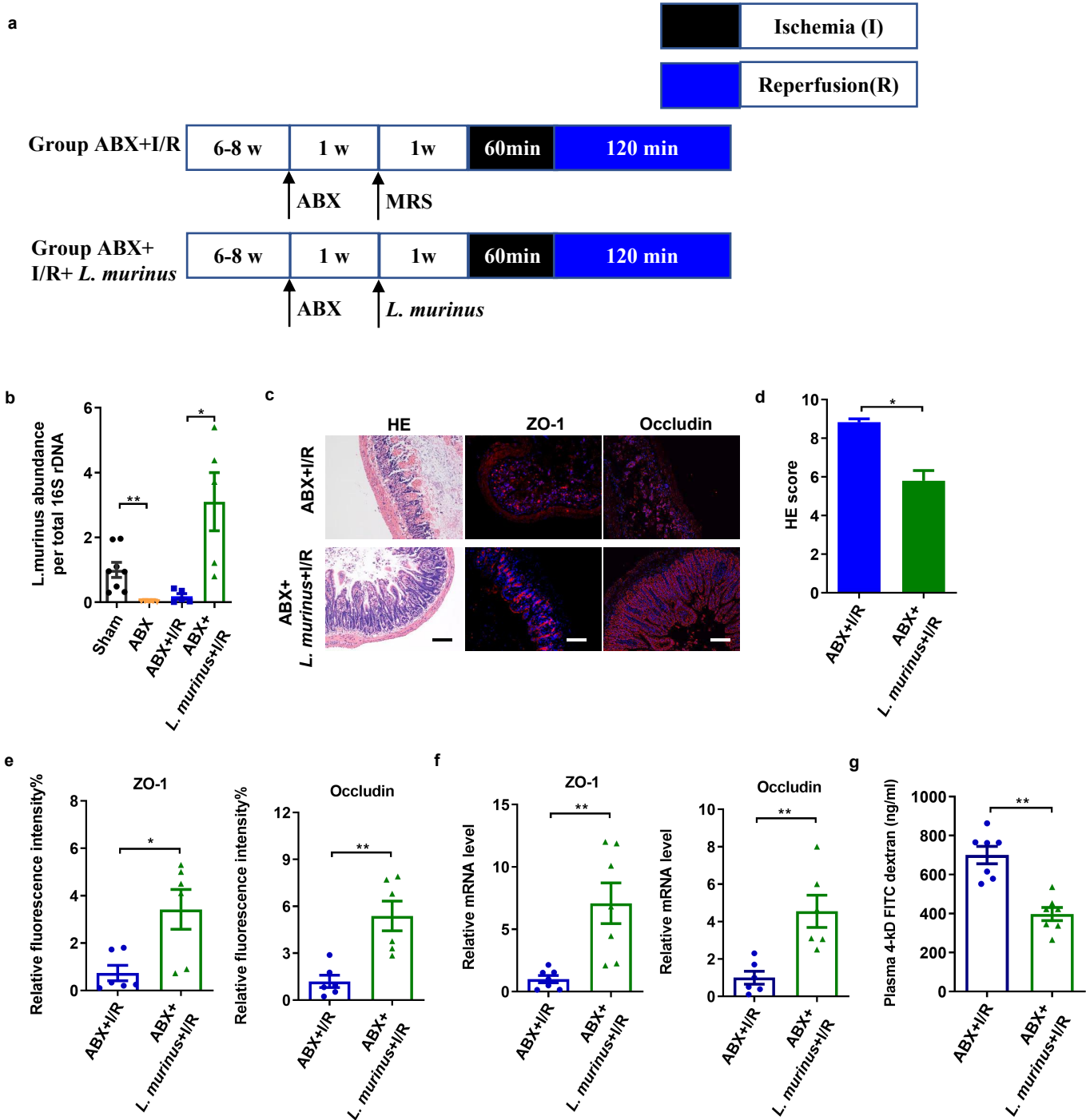

Supplement: Supplementary file 6 — Additional file 5: Figure S4. Lactobacillus murinus improves intestinal I/R-induced intestinal injury in antibiotic-treated mice. a. Experimental design. The WT mice were randomly divided into the following groups. Group ABX + I/R, in which ABX was administered 1w before intestinal ischemia; Group ABX + I/R + L. murinus, treatment of mice with ABX for a week, and then mice were gavaged daily for 7 days with L. murinus. b Relative abundance of L. murinus in the cecum after ABX clearance experiment. c-e HE staining and ZO-1 and occludin immunofluorescent staining in the ileum. Representative quantification on the right. Scale bar=100 μm. f Tight junction mRNA levels in the ileum. g FD-4 level in the plasma. The results are expressed as the mean ± SEM. n = 8. ***p<0.001, **p<0.01, *p<0.05 were determined by two-tailed Student’s t-test. ABX, antibiotic; FD-4, FITC Dextran 4-KD; I/R, ischemia/reperfusion; HE, hematoxylin-eosin; L. murinus, Lactobacillus murinus; SMA, superior mesenteric artery. [file 40168_2022_1227_MOESM6_ESM.pdf]

Figure S5

a

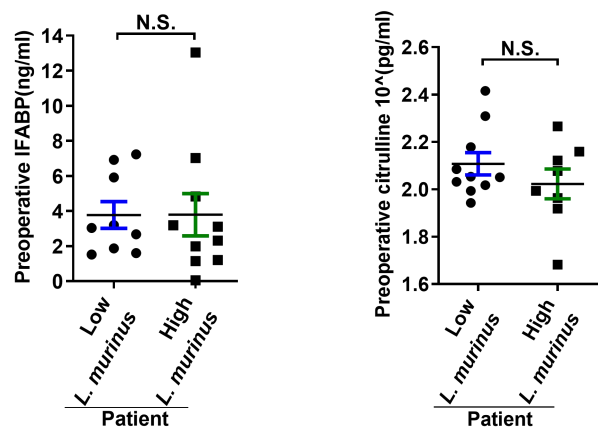

Supplement: Supplementary file 8 — Additional file 7: Figure S5. The level of IFABP and citrulline in serum before surgery between low L. murinus abundance group and high L. murinus abundance group. [file 40168_2022_1227_MOESM8_ESM.pdf]

Figure abstract

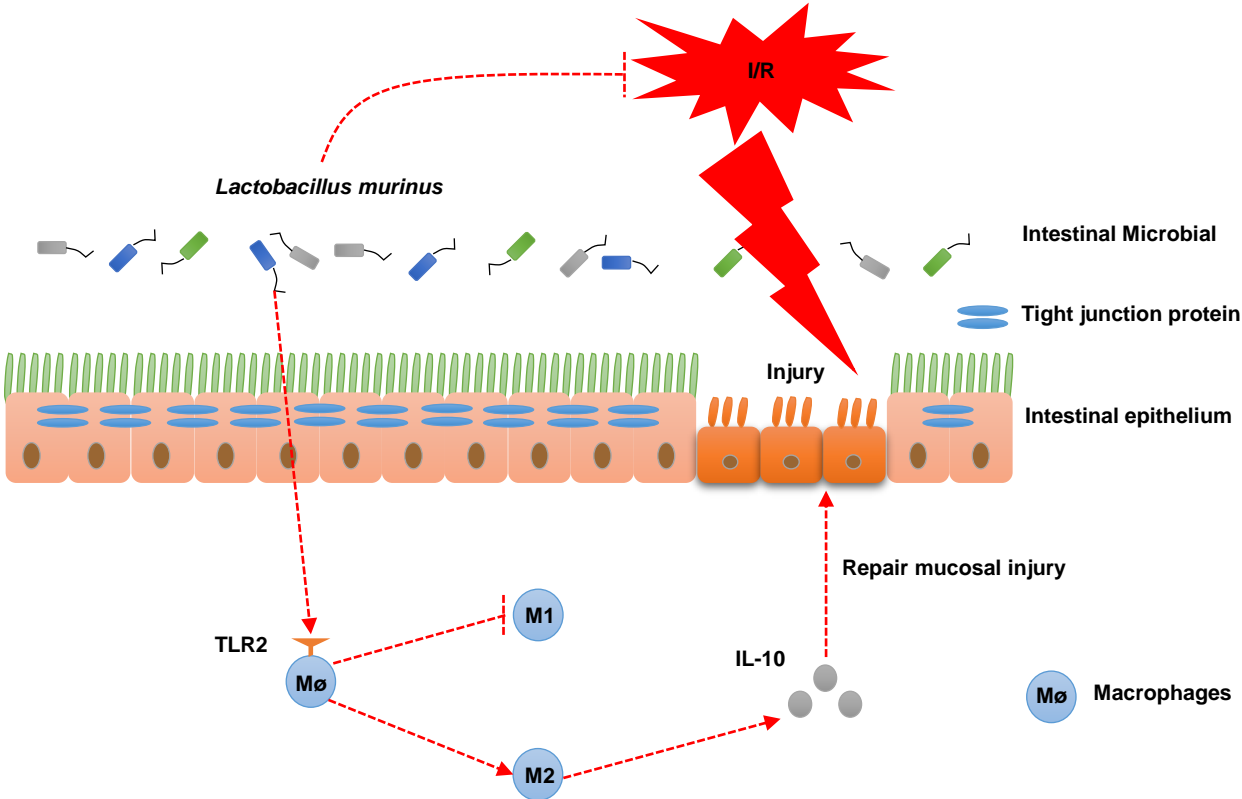

Supplement: Supplementary file 9 — Additional file 8. Figure abstract. [file 40168_2022_1227_MOESM9_ESM.pdf]
