# Supplementary material for: Lactobacillus murinus alleviate intestinal ischemia/reperfusion injury through promoting the release of interleukin-10 from M2 macrophages via Toll-like receptor 2 signaling
Source: Microbiome. 2022 Mar 3;10:38. doi: 10.1186/s40168-022-01227-w (PMC8896269; doi:10.1186/s40168-022-01227-w)
Supplement: Supplementary file 4 — Additional file 3: Table S1. Primers sequence. [file 40168_2022_1227_MOESM4_ESM.pdf]

Supplementary table1 primer Sequences

Gene	Forward primer (5'-3')	Reverse primer (5'-3')
18S	CGATCCGAGGGCCTCACTA	AGTCCCTGCCCTTTGTACACA
IL-1β	TGTGAAATGCCACCTTTTGA	GGTCAAAGGTTTGGAAGCAG
IL-6	TGATGCACTTGCAGAAAACA	ACCAGAGGAAATTTTCAATAGGC
TNFα	CCACCACGCTCTTCTGTCTAC	AGGGTCTGGGCCATAGAACT
MPO	GATGACCCCTGCCTCCTC	GCTCTCGAACAAAGAGGGTG
Claudin-1	GAGGGACTGTGGATGTCCTG	ATGCCAATTACCATCAAGGC
Claudin-2	CAGAGCTCTTCGAAAGGACG	TCACAGTGTCTCTGGCAAGC
Claudin-7	TGTACAAGGGGCTCTGGATG	GGACACCACCATTAAGGCTC
Occludin	CATTTATGATGAACAGCCCC	GGACTGTCAACTCTTTCCGC
Tjp-1	AGAGACAAGATGTCCGCCAG	TGCAATTCCAAATCCAAACC
Tjp-2	GTGATTTTCTTCAACCCGGA	TTTTTGAGCTTGTTGGCTTG
Cxcl2	CGGTCAAAAAGTTTGCCTTG	TCCAGGTCAGTTAGCCTTGC
Ccl3	ACCATGACACTCTGCAACCA	GTGGAATCTTCCGGCTGTAG
Myd88	TCATGTTCTCCATACCCTTGGT	AAACTGCGAGTGGGGTCAG
16S	GTGSTGCAYGGYTGTCGTCA	ACGTCRTCCMCACCTTCCTC
Firmicutes	GGAGYATGTGGTTTAATTCGA	AGCTGACGACAACCATGCAC
Bacteroidetes	GGCGACCGGCGCACGGG	GRCCTTCCTCTCAGAACCC
