# Supplementary material for: Lactobacillus murinus alleviate intestinal ischemia/reperfusion injury through promoting the release of interleukin-10 from M2 macrophages via Toll-like receptor 2 signaling
Source: Microbiome. 2022 Mar 3;10:38. doi: 10.1186/s40168-022-01227-w (PMC8896269; doi:10.1186/s40168-022-01227-w)
Supplement: Supplementary file 7 — Additional file 6: Table S2. Characteristics of patients with cardiopulmonary bypass. [file 40168_2022_1227_MOESM7_ESM.pdf]

Supplementary table2 patient characteristics

Parameter	Low *L. murinus* patient(n=10)	High *L. murinus*	*P*
male	4(40)	3(30)	0.66
Age, yr	59(56-61)	50(36-61)	0.1
Body mass index, kg m^{-2}	23.3(20.8-25.1)	23.7(21.5-24.8)	0.66
Duration of anesthesia, mir	295(283-338)	288(280-370)	0.83
Duration of surgery, min	235(226-290)	236(223-309)	0.77
Duration of CPB, min	110(101-134)	118(93-169)	0.83
Estimated blood loss, ml	300(300-375)	400(300-400)	0.2

Note. Numbers are counts (percentage) or median (interquartile range).
